# Couples’ experiences with ostomy due to colorectal cancer in one of the partners: a scoping review

**DOI:** 10.1590/1980-220X-REEUSP-2025-0250en

**Published:** 2026-02-23

**Authors:** Ricardo Emanuel Sousa Mestre, Ana Beatriz Gomes Dias, Célia Maria Gonçalves Simão de Oliveira, Patrícia Vinheiras Alves

**Affiliations:** 1 Escola Superior de Saúde Atlântica, Lisboa, Portugal.; 2 Instituto Português de Oncologia de Lisboa Francisco Gentil, Lisboa, Portugal.; 3 Universidade de Lisboa, Escola Superior de Enfermagem, Lisboa, Portugal.; 4 Universidade de Lisboa, Escola Superior de Enfermagem, Centro de Investigação Inovação e Desenvolvimento em Enfermagem de Lisboa, Lisboa, Portugal.

**Keywords:** Adaptation, Psychological, Social Adjustment, Colorectal Neoplasms, Family Characteristics, Ostomy

## Abstract

**Objective:**

To map scientific evidence on the shared experience of couples undergoing intestinal ostomy for colorectal cancer.

**Method:**

A scoping review was conducted in accordance with JBI guidelines. The search was performed in 13 databases and repositories, with inclusion criteria defined by the PCC (Participants, Concept, and Context) approach.

**Results:**

Nine studies were included, which allowed mapping couples’ experiences and summarizing its characteristics according to the nature, influencing factors, and results arising from this experience; in naming this experience, the concepts of adaptation and/or adjustment predominated.

**Conclusion:**

The experience of couples with ostomies due to colorectal cancer varies among them, involving physical, emotional, and relational challenges. Gaps in scientific evidence have been identified, highlighting the need for primary research to better understand this experience, particularly the dynamics or processes experienced by these couples.

## INTRODUCTION

Globally, colorectal cancer (CRC) was the third most commonly diagnosed type of cancer in 2020, ranking third in incidence and second in mortality^(1)^. Despite its seriousness, in recent years there has been a reduction in the mortality rate from CRC, attributed to advances in early diagnosis and treatment^(2)^. Surgery is the treatment of choice^(3,4,5)^, frequently resulting in the construction of a temporary or permanent bowel elimination ostomy (BEO)^(6)^. Although precise global data is not available, it is estimated that around 1 million people in the United States^(7,8)^, 1 million in China and 700,000 in Europe currently live with at least one ostomy^(7)^, and this number is expected to continue to increase as a result of the growing incidence of CRC^(9)^.

CRC diagnosis and treatment represent a profoundly challenging experience, marked by physical and emotional suffering, sadness, anxiety, fear, and insecurity^(10)^. The presence of a BEO exacerbates the changes experienced by a person, generating additional challenges related to body image, self-esteem, sexuality, and participation in social, physical, and leisure activities^(11,12)^. These changes directly affect daily life and perceived quality of life^(11,12,13)^.

Starting from the principle that events experienced by one family member affect the others^(14)^, it is plausible to affirm that, in the context of a marital relationship, these changes are experienced by the couple as a unit. A couple is considered to be the union of two adults who maintain a significant, intimate and lasting relationship^(15)^, sharing a common life project^(16)^. In this context, a spouse often takes over primary caregiver, also facing substantial changes in their daily life, with emotional, social, and relational repercussions^(17,18)^. Thus, it is understood that the experience lived by couples in response to CRC and BEO is reflected in joint readjustments, with a significant impact on the marital relationship, as well as on spouses’ mental and physical health^(18,19,20)^.

The experience of a couple facing a challenge of this nature is recognized as a dynamic and evolving process, characterized by continuous transitions^(20)^, mutual adaptations^(20,21,22)^ and marital adjustments^(20,23)^. This perspective underscores that couples’ experiences are constantly transforming, reflected in how spouses adapt and reorganize themselves in the face of changes imposed by the health condition.

In this context, the changes brought about by CRC and BEO trigger a process that can be conceptualized as transition, understood as the passage from one condition or state to another^(24,25)^. Transition, as a dynamic human experience, requires individuals, or in this case the couple, to engage in processes of adaptation and adjustment^(24,25,26)^. This process is complex and occurs throughout the life cycle, being triggered by critical events – particularly those related to health and role performance – and resulting in role changes, acquisition of new knowledge, changes in those roles, and adaptation of behaviors^(25,27)^.

Thus, adaptation and adjustment emerge as central elements of that process, influencing couples’ ability to reorganize their lives in light of a new situation^(27,28)^. Adaptation refers to the ability to reorganize one’s life and identity, integrating the new demands imposed by the health condition, and promoting well-being, marital relationship continuity, and the relationship with the outside world^(29)^. Adjustment, in turn, refers to couples’ active involvement, and is conditioned by the knowledge that both have about their own situation and by their ability to manage changes and integrate into the new life condition^(27,30)^. Thus, adaptation translates to integration of changes into daily life, while adjustment reflects the ongoing process of managing and coping with these changes.

Couples facing BEO due to CRC constitute a particular group with regard to healthcare needs^(17)^. However, scientific evidence has predominantly focused on the individual experience of a person with BEO, the spouse as a caregiver, or the family in general, neglecting the understanding of couples’ shared experience as a unit. This gap is particularly relevant, since the experience of BEO due to CRC is not limited to affected individuals, but encompasses the couple, influencing their relationship, communication, roles, and emotional well-being. Nursing, as a discipline that prioritizes person-centered care, has a responsibility to understand this shared experience, promoting interventions that respond to couples’ needs as a unit^(27,31,32)^. Preliminary research in the JBI Evidence Synthesis, Cochrane, CINAHL, MEDLINE, PROSPERO, Open Science Framework, and Figshare databases, using the search terms “transition”, “adaptation”, “adjustment”, “colorectal neoplasms”, “ostomy”, and “couple”, in Portuguese, English, French, and Spanish, did not identify any published or ongoing literature review that focuses on the couple as a participating unit in the process of experiencing a BEO due to CCR.

In this regard, this scoping review is justified, which aims to map^(33,34,35)^the scientific evidence on the joint experience of couples facing BEO due to CRC, identifying available scientific evidence and gaps in this knowledge, with the aim of providing a basis for future research that contributes to the development of nursing knowledge regarding this phenomenon in this population.

## METHOD

### Study Design

This is a scoping review carried out according to the methodology proposed by JBI^(33,34)^ and the Preferred Reporting Items for Systematic Reviews and Meta-Analyses extension for Scoping Reviews (PRISMA-ScR) checklist guidelines^(34,36)^. The protocol was previously registered on the Open Science Framework platform, with the following DOI: 10.17605/OSF.IO/E8WJD. During the review process, it became necessary to introduce changes to the protocol: the terminology used in the title, objective, and review questions was reformulated, due to the finding that the selected articles did not use the concept of transition to name couples’ experiences.

The review question was defined as “What scientific evidence is available on the shared experience of couples facing a BEO due to CRC?”, as well as sub-questions “What studies exist on couples’ experiences facing a BEO due to CRC?”, “What are the characteristics of the population in the available evidence on this experience?”, “What concepts are used to describe couples’ experiences?” and “How are couples’ experiences characterized in the available evidence?”.

### Selection Criteria

Eligibility criteria were defined based on the PCC mnemonic (Participants, Concept, and Context), as described by Peters *et al.* ^(33,34)^. Concerning participants, the review considered studies that included adults (aged ≥ 19 years) in a marital relationship, where one of the partners had a BEO due to CRC, and where data were collected from the couple, the person with the ostomy, or the spouse. For eligibility purposes, “couple” was defined as any explicit reference in the studies to terms such as “couple”, “partner”, “spouse”, or “husband/wife”, regardless of gender or marital status. Studies in which BEO was not constructed following CRC were excluded.

As for the concept, studies that addressed couples’ experiences with BEO resulting from CRC were included, as well as studies that investigated couples’ transition, adaptation, or adjustment to this condition. Studies that did not focus on couples’ experiences, transition, adjustment, or adaptation were excluded, as were those that explored these concepts only at an individual level, focusing exclusively on a person with an ostomy or their spouse. As for the context, no restrictions related to cultural, ethnic, gender, or geographic location factors were imposed.

The review included primary and secondary studies, and one thesis. Literature reviews were identified in the selected studies. However, no data duplication was found resulting from the inclusion of primary studies in this review; therefore, there was no need to exclude these primary studies^(34)^. Due to the purpose of the review, books, book chapters, and opinion articles were excluded. Studies published in English, Portuguese, French, and Spanish were considered, with no time restrictions applied.

### Data Collection

An initial search was conducted between May and June 2024, and updated in July 2025, following a multi-phase approach, with the aim of identifying as many relevant studies and publications as possible. In the first phase, searches were carried out in the MEDLINE (via EBSCO), CINAHL (via EBSCO), SciELO, Cochrane Database of Systematic Reviews (via EBSCO), JBI Evidence Synthesis, Psychology and Behavioral Sciences Collection (via EBSCO), PsycINFO (via EBSCO), Web of Science, and Scopus electronic databases. In the second phase, with the aim of integrating grey literature, searches were conducted in the Open Access Theses and Dissertations, ProQuest Dissertation and Theses Database, *Repositório Científico de Acesso Aberto de Portugal* (RCAAP), and Google Scholar databases. It was not possible to include data from OpenGrey and DART-Europe, as they were inactive at the time of data collection. Finally, in the third phase, the bibliographic references of included studies were analyzed to identify additional articles potentially relevant to the study.

The previously identified Medical Subject Headings were included and adapted for each information source used, developing a comprehensive search strategy with appropriate truncation operators and Booleans (Chart 1).

**Chart 1:** Search strategy – Lisbon, Portugal, 2025.

Database	Search strategy
CINAHL complete (EBSCO)	(XB (caregiv* OR “care partner” OR conjugal OR coupl* OR dyad* OR “family member” OR husband* OR “life partner” OR marital* OR married* OR mutuality OR partner* OR spous* OR wife OR wive) OR MH (caregivers OR marriage OR “married women” OR “married men” OR spouses OR “significant other”)) AND (XB (stoma* OR enterostomy OR ostomy OR ileostomy OR colostomy) OR MH ostomy+) AND (XB (“colorectal cancer” OR “CRC” OR “bowel cancer” OR “colon cancer” OR “rectal cancer”) OR MH (“cancer patients” OR “intestinal neoplasm+” OR neoplasms)) AND (XB (Change* OR experience* OR “life-change event” OR “life changing event” OR “life-changing event” OR “life change*” OR transition* OR adjustment* OR adaptation) OR MH (“life change events+” OR “adaptation, psychological+” OR “life experiences+” OR “social adjustment”))
MEDLINE Complete (EBSCO)	(XB (caregiv* OR “care partner” OR conjugal OR coupl* OR dyad* OR “family member” OR husband* OR “life partner” OR marital* OR married* OR mutuality OR partner* OR spous* OR wife OR wive) OR MH (caregivers OR marriage OR spouses)) AND (XB (stoma* OR enterostomy OR ostomy OR ileostomy OR colostomy) OR MH (ostomy+ OR “surgical stomas”)) AND (XB (“colorectal cancer” OR “CRC” OR “bowel cancer” OR “colon cancer” OR “rectal cancer”) OR MH (“intestinal neoplasms+” OR neoplasms)) AND (XB (Change* OR experience* OR “life-change event” OR “life changing event” OR “life-changing event” OR “life change*” OR transition* OR adjustment* OR adaptation) OR MH (“life change events” OR “adaptation, psychological+” OR “social adjustment”))
Psychology & Behavioral Sciences Collection (EBSCO)	(XB (caregiv* OR “care partner” OR conjugal OR coupl* OR dyad* OR “family member” OR husband* OR “life partner” OR marital* OR married* OR mutuality OR partner* OR spous* OR wife OR wive) OR SU (caregiv* OR “care partner” OR conjugal OR coupl* OR dyad* OR “family member” OR husband* OR “life partner” OR marital* OR married* OR mutuality OR partner* OR spous* OR wife OR wive)) AND (XB (stoma* OR enterostomy OR ostomy OR ileostomy OR colostomy) OR SU (stoma* OR enterostomy OR ostomy OR ileostomy OR colostomy)) AND (XB (“colorectal cancer” OR “CRC” OR “bowel cancer” OR “colon cancer” OR “rectal cancer”) OR SU (“colorectal cancer” OR “CRC” OR “bowel cancer” OR “colon cancer” OR “rectal cancer”)) AND (XB (Change* OR experience* OR “life-change event” OR “life changing event” OR “life-changing event” OR “life change*” OR transition* OR adjustment* OR adaptation) OR SU (Change* OR experience* OR “life-change event” OR “life changing event” OR “life-changing event” OR “life change*” OR transition* OR adjustment* OR adaptation))
PsycInfo (APA PsycNet)	(title: (caregiv*) OR title: (“care partner”) OR title: (conjugal) OR title: (coupl*) OR title: (dyad*) OR title: (“family member”) OR title: (husband*) OR title: (“life partner”) OR title: (marital*) OR title: (married*) OR title: (mutuality) OR title: (partner*) OR title: (spous*) OR title: (wife) OR title: (wive)) OR (abstract: (caregiv*) OR abstract: (“care partner”) OR abstract: (conjugal) OR abstract: (coupl*) OR abstract: (dyad*) OR abstract: (“family member”) OR abstract: (husband*) OR abstract: (“life partner”) OR abstract: (marital*) OR abstract: (married*) OR abstract: (mutuality) OR abstract: (partner*) OR abstract: (spous*) OR abstract: (wife) OR abstract: (wive)) OR (MeSH: (Caregivers) OR MeSH: (marriage) OR MeSH: (spouses))) AND ((title: (caregiv*) OR title: (“care partner”) OR title: (conjugal) OR title: (coupl*) OR title: (dyad*) OR title: (“family member”) OR title: (husband*) OR title: (“life partner”) OR title: (marital*) OR title: (married*) OR title: (mutuality) OR title: (partner*) OR title: (spous*) OR title: (wife) OR title: (wive)) OR (abstract: (caregiv*) OR abstract: (“care partner”) OR abstract: (conjugal) OR abstract: (coupl*) OR abstract: (dyad*) OR abstract: (“family member”) OR abstract: (husband*) OR abstract: (“life partner”) OR abstract: (marital*) OR abstract: (married*) OR abstract: (mutuality) OR abstract: (partner*) OR abstract: (spous*) OR abstract: (wife) OR abstract: (wive)) OR (MeSH: (Caregivers) OR MeSH: (marriage) OR MeSH: (spouses)) AND (title: (stoma* OR enterostomy OR ostomy OR ileostomy OR colostomy)) OR (abstract: (stoma* OR enterostomy OR ostomy OR ileostomy OR colostomy)) OR (MeSH: (Ostomy) OR MeSH: (“surgical stomas”)) AND (title: (“colorectal cancer”) OR title: (“CRC”) OR title: (“bowel cancer”) OR title: (“colon cancer”) OR title: (“rectal cancer”)) OR (abstract: (“colorectal cancer”) OR abstract: (“CRC”) OR abstract: (“bowel cancer”) OR abstract: (“colon cancer”) OR abstract: (“rectal cancer”)) OR (MeSH: (“intestinal neoplasms”) OR MeSH: (neoplasms)) AND (title: (Change*) OR title: (experience*) OR title: (“life-change event”) OR title: (“life changing event”) OR title: (“life-changing event”) OR title: (“life change*”) OR title: (transition*) OR title: (adjustment*) OR title: (adaptation)) OR (abstract: (Change*) OR abstract: (experience*) OR abstract: (“life-change event”) OR abstract: (“life changing event”) OR abstract: (“life-changing event”) OR abstract: (“life change*”) OR abstract: (transition*) OR abstract: (adjustment*) OR abstract: (adaptation)) OR (MeSH: (“life change events”) OR MeSH: (“adaptation, psychological”) OR MeSH: (“social adjustment”))
Scopus	(TITLE-ABS-KEY (caregiv* OR “care partner” OR conjugal OR coupl* OR dyad* OR “family member” OR husband* OR “life partner” OR marital* OR married* OR mutuality OR partner* OR spous* OR wife OR wive )) AND ( TITLE-ABS-KEY ( stoma*)) AND (TITLE-ABS-KEY ( “colorectal cancer” OR “CRC” OR “bowel cancer” OR “colon cancer” OR “rectal cancer”)) AND (TITLE-ABS-KEY (Change* OR experience* OR “life-change event” OR “life changing event” OR “life-changing event” OR “life change*” OR transition* OR adjustment* OR adaptation))
Web of Science Core Collection (Web of Science)	(TS=(caregiv* OR “care partner” OR conjugal OR coupl* OR dyad* OR “family member” OR husband* OR “life partner” OR marital* OR married* OR mutuality OR partner* OR spous* OR wife OR wive)) AND (TS=(stoma* OR enterostomy OR ostomy OR ileostomy OR colostomy)) AND (TS=(“colorectal cancer” OR “CRC” OR “bowel cancer” OR “colon cancer” OR “rectal cancer”)) AND (TS=(Change* OR experience* OR “life-change event” OR “life changing event” OR “life-changing event” OR “life change*” OR transition* OR adjustment* OR adaptation))
SciELO Citation Index (Web of Science)	(TS=(caregiv* OR “care partner” OR conjugal OR coupl* OR dyad* OR “family member” OR husband* OR “life partner” OR marital* OR married* OR mutuality OR partner* OR spous* OR wife OR wive)) AND (TS=(stoma* OR enterostomy OR ostomy OR ileostomy OR colostomy)) AND (TS=(“colorectal cancer” OR “CRC” OR “bowel cancer” OR “colon cancer” OR “rectal cancer”)) AND (TS=(Change* OR experience* OR “life-change event” OR “life changing event” OR “life-changing event” OR “life change*” OR transition* OR adjustment* OR adaptation))
Cochrane	(caregiv* OR “care partner” OR conjugal OR coupl* OR dyad* OR “family member” OR husband* OR “life partner” OR marital* OR married* OR mutuality OR partner* OR spous* OR wife OR wive):ti,ab,kw MeSH descriptor: [Caregivers] explode all trees MeSH descriptor: [Marriage] explode all trees MeSH descriptor: [Spouses] explode all trees #1 OR #2 OR #3 OR #4 (stoma* OR enterostomy OR ostomy OR ileostomy OR colostomy):ti,ab,kw MeSH descriptor: [Ostomy] explode all trees MeSH descriptor: [Surgical Stomas] explode all trees #6 OR #7 OR #8 (“colorectal cancer” OR “CRC” OR “bowel cancer” OR “colon cancer” OR “rectal cancer”):ti,ab,kw MeSH descriptor: [Intestinal Neoplasms] explode all trees MeSH descriptor: [Neoplasms] explode all trees #10 OR #11 OR #12 (Change* OR experience* OR “life-change event” OR “life changing event” OR “life-changing event” OR life NEXT change* OR transition* OR adjustment* OR adaptation):ti,ab,kw MeSH descriptor: [Life Change Events] explode all trees MeSH descriptor: [Adaptation, Psychological] explode all trees MeSH descriptor: [Social Adjustment] explode all trees #14 OR #15 OR #16 OR #17 #5 AND #9 AND #13 AND #18
ProQuest Dissertations & Theses Citation Index (Web of Science)	(TS=(caregiv* OR “care partner” OR conjugal OR coupl* OR dyad* OR “family member” OR husband* OR “life partner” OR marital* OR married* OR mutuality OR partner* OR spous* OR wife OR wive)) AND (TS=(stoma* OR enterostomy OR ostomy OR ileostomy OR colostomy)) AND (TS=(“colorectal cancer” OR “CRC” OR “bowel cancer” OR “colon cancer” OR “rectal cancer”)) AND (TS=(Change* OR experience* OR “life-change event” OR “life changing event” OR “life-changing event” OR “life change*” OR transition* OR adjustment* OR adaptation))
RCAAP (EBSCO)	(TI (caregiv* OR “care partner” OR conjugal OR coupl* OR dyad* OR “family member” OR husband* OR “life partner” OR marital* OR married* OR mutuality OR partner* OR spous* OR wife OR wive) OR TI (caregiv* OR “care partner” OR conjugal OR coupl* OR dyad* OR “family member” OR husband* OR “life partner” OR marital* OR married* OR mutuality OR partner* OR spous* OR wife OR wive) OR SU (caregiv* OR “care partner” OR conjugal OR coupl* OR dyad* OR “family member” OR husband* OR “life partner” OR marital* OR married* OR mutuality OR partner* OR spous* OR wife OR wive)) AND (TI (stoma* OR enterostomy OR ostomy OR ileostomy OR colostomy) OR AB (stoma* OR enterostomy OR ostomy OR ileostomy OR colostomy) OR SU (stoma* OR enterostomy OR ostomy OR ileostomy OR colostomy)) AND (TI (“colorectal cancer” OR “CRC” OR “bowel cancer” OR “colon cancer” OR “rectal cancer”) OR AB (“colorectal cancer” OR “CRC” OR “bowel cancer” OR “colon cancer” OR “rectal cancer”) OR SU (“colorectal cancer” OR “CRC” OR “bowel cancer” OR “colon cancer” OR “rectal cancer”)) AND (TI (Change* OR experience* OR “life-change event” OR “life changing event” OR “life-changing event” OR “life change*” OR transition* OR adjustment* OR adaptation) OR AB (Change* OR experience* OR “life-change event” OR “life changing event” OR “life-changing event” OR “life change*” OR transition* OR adjustment* OR adaptation) OR SU (Change* OR experience* OR “life-change event” OR “life changing event” OR “life-changing event” OR “life change*” OR transition* OR adjustment* OR adaptation)) Filters: Content Provider: RCAAP
JBI Evidence Synthesis	stoma* OR enterostomy OR ostomy OR ileostomy OR colostomy
Google Scholar	allintitle: stoma cancer experience spouse allintitle: stoma cancer transition spouse allintitle: stoma cancer adaptation spouse allintitle: stoma cancer adjustment spouse allintitle: ostomy cancer experience spouse allintitle: ostomy cancer transition spouse allintitle: ostomy cancer adaptation spouse allintitle: ostomy cancer adjustment spouse allintitle: *ostomia cancro experiência casal* allintitle: *ostomia cancro transição casal* allintitle: *ostomia cancro adaptação casal* allintitle: *ostomia cancro ajustamento casal*
Open Access Thesis and Dissertations	abstract (caregiv* OR partner OR conjugal OR coupl* OR dyad* OR family OR husband* OR marital* OR married* OR spous* OR wife OR wive) AND abstract (stoma* OR enterostomy OR ostomy OR ileostomy OR colostomy) AND abstract (cancer* OR neoplasm*) AND abstract (Change* OR experience* OR transition* OR adjustment* OR adaptation) subject (caregiv* OR partner OR conjugal OR coupl* OR dyad* OR family OR husband* OR marital* OR married* OR spous* OR wife OR wive) AND subject (stoma* OR enterostomy OR ostomy OR ileostomy OR colostomy) AND subject (cancer* OR neoplasm*) AND subject (Change* OR experience* OR transition* OR adjustment* OR adaptation) title (caregiv* OR partner OR conjugal OR coupl* OR dyad* OR family OR husband* OR marital* OR married* OR spous* OR wife OR wive) AND title (stoma* OR enterostomy OR ostomy OR ileostomy OR colostomy) AND title (cancer* OR neoplasm*) AND title (Change* OR experience* OR transition* OR adjustment* OR adaptation)

The identified references were compiled and uploaded to the Rayyan^®^ platform, with duplicate records removed, resulting in a total of 733 documents. The analysis of document titles and abstracts was performed by two independent reviewers using the one-blind method. Discrepancies were resolved by a third reviewer^(33,36)^.

**Figure 1:**
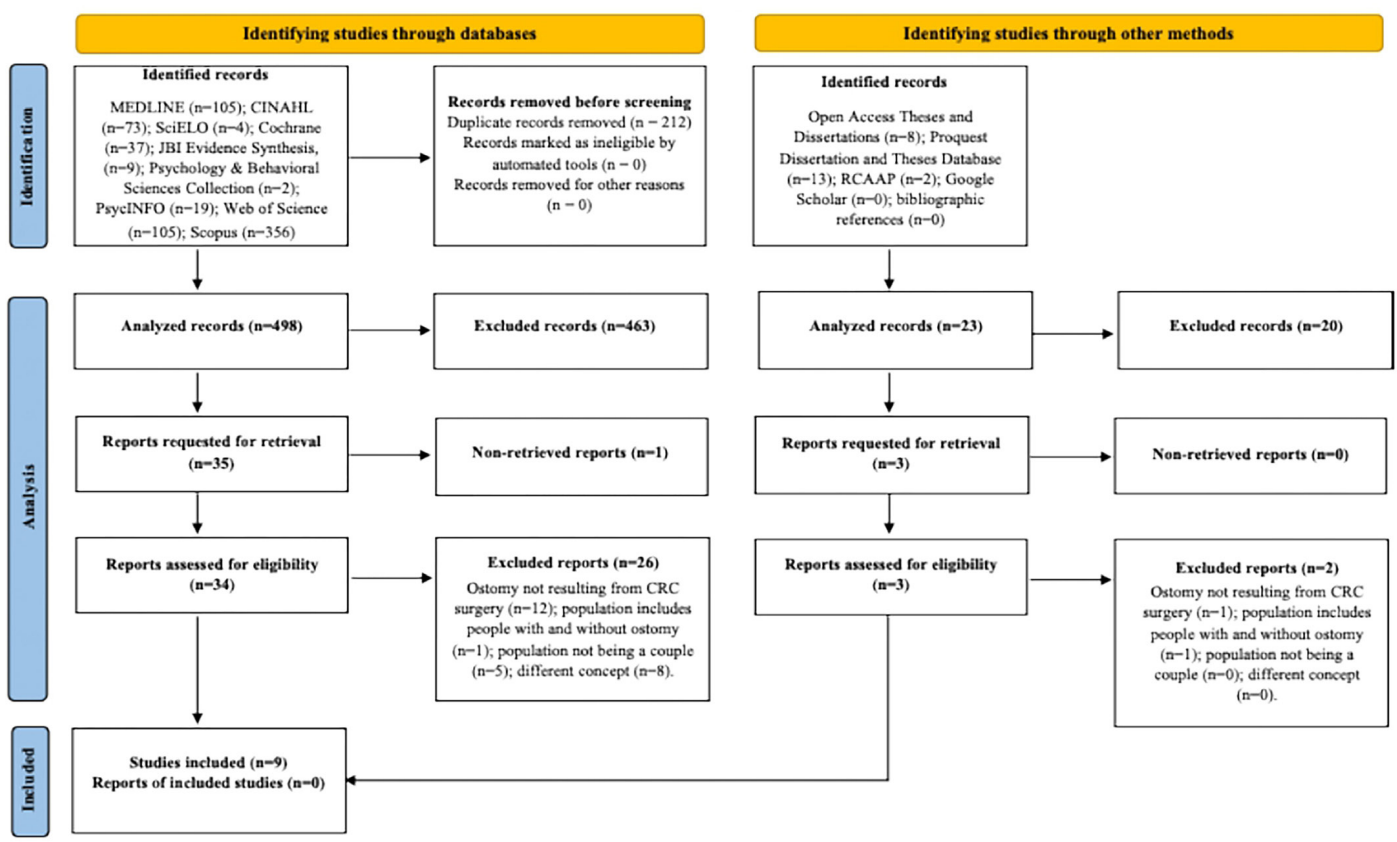
Preferred Reporting Items for Systematic Reviews and Meta-Analyses extension for Scoping Reviews flowchart diagram^(37)^.

Documents that met the inclusion criteria based on title and abstract analysis were retrieved in full for detailed analysis, with the complete text being read independently by reviewers. The document selection process is represented in the PRISMA-ScR flow diagram (Figure 1), which systematically illustrates process stages^(37)^, culminating in the selection of nine documents.

### Data Analysis and Treatment

Data extraction and presentation from included studies followed a structure specifically designed for this scoping review, based on the objective and review questions^(33,34)^. Data extraction was performed by two independent reviewers, and any discrepancies were resolved with the help of a third reviewer. Data related to the objective and review questions (Chart 2) were extracted, including title, author(s), year, country, study objective, methodology and methods, target population, and main results. The collected data were analyzed using frequency counting to characterize the included documents and descriptive qualitative content analysis to summarize the characteristics of couples’ experiences. The results are presented in a chart and through narrative description.

**Chart 2:** Extracted data presentation – Lisbon, Portugal, 2025.

	Author(s)/year/country	Methodology and study methods	Population	Objective	Key results
**Study 1**	Du, Chaiviboontha & Sumdaengrit/2024/China^(38)^	Qualitative paradigm Phenomenology Interview	16 people with ostomies (aged between 18 and 59 years)	Explore the experiences of colorectal cancer survivors in their marital intimacy after undergoing an ostomy.	The study identified five fundamental dimensions in the marital experience of colorectal cancer survivors with ostomies: physical, psychological, social, spiritual, and operational intimacy. The presence of an ostomy negatively affects sexual involvement, generating concerns about attractiveness and self-esteem, reflected in decreased libido and difficulties in affective expression. Even so, emotional and spiritual connections are perceived as particularly significant, contributing to a redefinition of identity and self-worth. Daily management (operational intimacy) emerges as a critical element, requiring collaboration, caregiver support, and conflict resolution skills to contribute to successful adaptation.
**Study 2**	Afiyanti, Fitch, Helen, Andjarwati, Rudi, Prawesti & Juliastuti/2023/ Indonesia^(39)^	Qualitative paradigm Phenomenology Interview	12 people with ostomies (aged between 31 and 58 years)	Explore the perspective of people living with ostomies regarding their sexual experiences and the physical, psychological, and cultural influences.	The study revealed significant sexual disturbances in colorectal cancer survivors with ostomies, including decreased desire, discomfort, and dissatisfaction, leading some to completely interrupt sexual intimacy. Spousal support emerged as a positive factor, promoting better communication and greater availability for companionship, recognized as beneficial by participants. Many coping strategies focused on marital well-being, sometimes at the expense of individual sexual needs. However, a gap in communication about sexual issues with healthcare professionals was observed. The results underscore the need for interdisciplinary support and pre- and post-surgical counseling to improve sexual health and marital relationships.
**Study 3**	Du, Wang, Du, Zou & Jin/2021/China^(40)^	Quantitative paradigm Correlational study One questionnaire and three scales	390 people with ostomies (59.2% were men; and 60.2% of them were over 60 years old)	Assess the correlation between intimate relationship with the spouse, self-disclosure, and adaptability in patients with colorectal cancer and enterostomy.	The study highlights acceptance as the dimension most valued by participants. On the other hand, self-disclosure was also positively correlated with levels of adaptation, acceptance, and a positive attitude towards life, while it correlated negatively with ongoing worry. The majority of participants (42.6%) reported problems in their marital intimate relationships, and 90.8% presented low or moderate levels of adaptation. Significant predictors for marital intimate relationships included educational background, self-care capacity, and postoperative time. The study emphasizes the need for health education to improve self-disclosure and adaptability among patients with colorectal cancer.
**Study 4**	McCarthy/2020/ Canada^(41)^	Qualitative paradigm Study 1 – Grounded Theory (semi-structured interview with couples) Only study 1 meets the inclusion criteria	11 couples (ostomy patient aged between 42 and 80 years; spouse aged between 37 and 76 years)	Provide an experiential account of couples’ sexual and intimate adjustment to a permanent colostomy (following treatment for colorectal cancer).	The results highlight significant emotional distress and identity concerns in people with colorectal cancer, reinforcing the importance of proactive supportive care from healthcare professionals to improve their quality of life. Despite the impact of cancer treatments and ostomy on sexual functioning and body image, couples demonstrate the ability to remain united as a dyad and to withstand the resulting changes. Although some report a strengthening of intimacy, physical, and psychosocial challenges persist in sexual adaptation. The results were presented in two categories. In the “Loving with an imperfect body after a colostomy” category, the importance of body image reconstruction as a facilitator of body acceptance and integration of an ostomy into one’s identity, the partner’s (dis)comfort, and the influence of mutual acceptance on openness to sexual intimacy are highlighted. In the “Coping with sexual function after cancer” category, the implications of treatments on sexual function and identity, the individual rhythm of each member of the couple, the need to think holistically about joint communication, and the exploration of different forms of intimacy are highlighted.
**Study 5**	Tripaldi/2019/ Ireland^(42)^	Literature review	18 articles - two literature reviews and the rest were research studies (qualitative, quantitative and mixed studies)	Explore the sexual experience of women after the formation of a ostomy during colorectal cancer surgery.	The review identified three main topics—disturbance of body image, intimate relationships, and devaluation of sexuality—highlighting the inadequacy of preoperative education in the sexual health area. Women reported feeling unprepared for the impact of an ostomy, facing difficulties in sexual function and in acceptance by their partner. The presence of an ostomy accentuates changes in body image, contributing to a reduction in self-esteem and perception of sexual attractiveness, associated with loss of libido, vaginal dryness, and limitations in participation in penetrative sexual relations. Women tend to anticipate acceptance or rejection from their spouse, and marital difficulties are also reported. Partner support proved essential for women’s adjustment, although not all partners were able to offer adequate emotional support. Thus, the importance of an integrated educational and psychosocial intervention in the postoperative period becomes evident as a fundamental strategy to promote the well-being of these women.
**Study 6**	Alwi, Setiawanb & Asrizalb/2018/ Indonesia^(43)^	Qualitative paradigm Phenomenology Interview	12 people with ostomies	Describe the experiences of a person with a permanent colostomy in relation to their quality of life.	The study identified seven central topics in the quality of life of people with permanent colostomies, namely: limitations in activities of daily living; restrictions in marital and social relationships; negative feelings towards the colostomy; financial difficulties; increasing demands of life; changes in rest needs and physical complications; and hope of living a normal life after the colostomy. In particular, marital relationships showed concerns regarding divorce and the cessation of sexual relations, generating fear and abstinence after surgery. In social interaction, anxiety, privacy, various practical difficulties, and introverted personality traits emerged as the main obstacles.
**Study 7**	Purba, Ibrahim & Rahayu/2018/ Indonesia^(44)^	Qualitative paradigm Phenomenology Interview	Three people with ostomies (aged between 40 and 50 years)	Gain meaningful experience from colorectal cancer survivors.	The study shows that colorectal cancer survivors with ostomy experience events that impact their marital relationship, particularly in the areas of intimacy, sexuality, mutual support, financial management, and loss. Self-esteem and body image are compromised, leading to shame, isolation, and difficulty in maintaining closeness with their partner. In the sexual realm, all reported changes in activity, marked by fear of pain, rejection, or discomfort, which requires greater professional support sensitive to the cultural context. The loss of physical integrity and the idealized future is faced with sadness, progressive acceptance, and a need for adaptation, often supported by faith. Despite the difficulties, the desire to help others in similar situations stands out, contributing to emotional strengthening.
**Study 8**	McCarthy, Fergus & Miller/2016/Canada^(45)^	Qualitative paradigm (theoretical thematic analysis) Interview	Nine couples (aged between 42 and 80 years)	Validate the Couple Adaptation to Cancer Classification System using a sample of couples who have adapted to colorectal cancer and permanent colostomy, and improve the framework based on emerging data from the sample.	The study found that all couples experienced fluctuations between “I” and “we” during the adaptation process to colorectal cancer and colostomy, highlighting different forms of affirmation and differentiation of “we”. While some participants identified clear changes in the relationship and agreed with the conceptualization that there is a continuous variation between bonding and separation, others showed a more mixed reaction, although they recognized the need for physical and emotional space throughout the course of the disease. Although respect for individual needs is not new in relational dynamics, several couples highlighted specific adjustments linked to colostomy activities and cancer treatment, such as reorganization of practical responsibilities. Additionally, some couples reported moments of heightened closeness and empathy, recognizing that these “union” phases can be interspersed with periods of distancing or isolation without definitively harming the marital bond. On the contrary, mutual recognition of independence and autonomy proved fundamental to strengthening collective and individual functioning. In summary, the dynamics of affirmation and erosion of the “we”, evidenced throughout the adjustment to illness, appear natural and do not necessarily constitute an indicator of relational failure. On the contrary, going through these challenges together reinforces couples’ resilience, intensifying sharing, empathy, and greater identification with the relationship.
**Study 9**	Persson, Severinsson & Hellström/2004/Sweden^(46)^	Qualitative paradigm (content analysis) Group interview	Nine spouses of people with ostomies (of whom six were women, aged between 49 and 74 years)	Understand spouses’ perceptions regarding how they live with a partner who has been diagnosed with colorectal cancer followed by surgery resulting in an ostomy.	The main findings of the study reveal that the experience of spouses of people undergoing ostomy for colorectal cancer is marked by a number of challenges. While spouses try to support their partners, they report difficulty in being involved in care and the information process, often feeling excluded and emotionally alone, as the focus of attention falls exclusively on the patient. Uncertainty about the disease’s progression and fears of cancer recurrence generated constant anxiety, exacerbated by a lack of information and the feeling of being excluded from clinical decisions. After surgery, it was necessary to learn to live in a new way, reorganizing the family routine, social habits, and care strategies according to ostomy demands, thus implying family adjustment and adaptation to the new condition. The presence of an ostomy had a significant impact on couples’ body image and intimacy, affecting sexuality and generating ambivalent feelings such as repulsion and guilt. Finally, spouses sought explanations for the origin of the disease, trying to find meaning in the suffering through the analysis of hereditary factors or the partner’s lifestyle.

## RESULTS

Through electronic searches in databases and repositories, 733 references were identified. Of these, 105 were found in the MEDLINE database, 73 in CINAHL, four in SciELO, nine in JBI Evidence Synthesis, two in Psychology and Behavioral Sciences Collection, 19 in PsycINFO, 105 in Web of Science, 356 in Scopus, 37 in Cochrane, eight in Open Access Theses and Dissertations, 13 in ProQuest Dissertation and Theses Database, and two in RCAAP. A search on Google Scholar did not return any relevant results.

Of the 733 records identified, 710 came from databases, and 23 resulted from other research methods. Regarding the records identified in the databases, 212 were excluded because they were duplicates. Of the remaining 498 records, 463 were eliminated after reading the title and abstract, and 35 were selected for full-text retrieval. Of these, one record was excluded due to the impossibility of accessing the full text, despite efforts made in this regard. A full reading of the remaining 34 records resulted in the exclusion of 26 because they did not meet the inclusion criteria, namely because the ostomy did not result from CRC surgery, because the population included both people with and without ostomies, because the population did not correspond to the couple, or because they addressed a different concept. In relation to the records identified by other methods, after reading the title and abstract, three were assessed according to inclusion criteria, with two being excluded because the ostomy did not result from CRC surgery and because the population included both people with and without ostomies. Thus, a study from other methods was included in the review. In total, nine studies were included in this review (Figure 1).

Included studies were published between 2004 and 2024, with at least one publication per year between 2019 and 2024^(38,39,40,41,42)^, except for 2022, in which no publications were recorded. There were also two publications in 2018^(43,44)^ and one publication each in 2016^(45)^ and 2004^(46)^. Regarding the geographical origin of the studies, three studies were found to have been carried out in Indonesia^(39,43,44)^, two in Canada^(41,45)^, two in China^(38,40)^, one in Ireland^(42)^, and one in Sweden^(46)^. Concerning the type of studies included, seven fall within the qualitative paradigm, with four of them using the phenomenological approach^(38,39,43,44)^, and one using Grounded Theory^(41)^, one using theoretically oriented thematic analysis^(45)^ and the other using content analysis^(46)^. One of the studies falls within the quantitative paradigm, of a correlational nature^(40)^, and one corresponds to a secondary study, in the form of a literature review^(42)^. With regard to the population, five studies included exclusively people with ostomies^(38,39,40,43,44)^, and two included couples^(41,45)^, in which one of the members had an ostomy, and one included spouses of people with ostomies^(46)^. Although the defined eligibility criteria considered adults to be people aged 19 or older, some studies consider adults to be people aged 18 or older. Thus, participants’ age ranged from 18 to 80 years.

Regarding the concepts used in the evidence accessed to describe couples’ experiences with BEO due to CRC, the concepts of adaptation and adjustment were identified.

A descriptive qualitative content analysis of results of included studies was performed. A meticulous and iterative data reading was conducted, from which topics characterizing couples’ experiences were inductively identified. These topics were subsequently grouped, by similarity, into categories and subcategories. The analysis process was conducted manually, without the use of specific software to support qualitative analysis, and was discussed among researchers until consensus was reached regarding the final definition and organization of categories.

The analysis of extracted data allowed us to characterize the experience of couples facing a BEO due to CRC in three categories: nature of the experience; influencing factors; and results arising from this experience (Chart 3).

**Chart 3:** Characterization of couples’ experiences – Lisbon, Portugal, 2025.

Dimensions		Data
**Nature of the experience**		A constant challenge as they deal with new and old physical and psychosocial concerns^(41,46)^ A feeling of disconnection, “in the same space, but not connected” to the partner^(45)^ Fluctuation between “I” and “we”^(45)^ Inevitability of periods of isolation from the spouse^(45)^ Various processes of affirmation and differentiation of the “we”^(45)^
**Factors**	**Facilitators**	Interdisciplinary support^(38-40,42)^ and pre- and post-surgical counseling^(39,40,42)^ Collaborative care^(38,39,45,46)^ and redistribution of responsibilities^(45,46)^ Support from the spouse^(39,41,42,46)^ Supportive care provided by healthcare professionals^(41)^ Self-care capacity^(40,42)^ Communication in couples^(39,41)^ and in society (community)^(41)^ Thinking in global terms^(41)^ Defining shared goals, genuine recognition, and encouragement^(38,46)^ Information, welcoming by healthcare professionals, and their responsiveness^(44)^ Positive attitude towards life^(40,46)^ Hope of living a normal life after ostomy^(43,46)^ Mutual trust^(38)^ Mutual understanding, empathy, and interpersonal experience^(45)^ Drawing on a sense of “we” to overcome adversity^(45)^ Non-sexual touch^(38)^ Contact with nature^(38)^ Stable socioeconomic status^(41)^ Faith^(38)^
	**Inhibitors**	Negative feelings regarding ostomy, such as fear^(39,43,44,46)^, insecurity^(43,44,46)^, anxiety^(38,43,46)^, depression^(38)^, shame^(38,43)^, concern^(38,40-44)^, psychological stress^(38,42)^ Lack of information and education about health^(38,40-42,46)^ Decreased libido^(38,39,42,43,46)^ Decreased attractiveness^(38,42,44,46)^ Lack of communication and support from professionals^(39,42)^ Lack of demonstrated interest in the spouse^(38)^ Financial difficulties^(43,44)^
**Results**	**Positives**	Acceptance of a new condition^(38,40-42,45,46)^ Improved communication within couples^(38,39,41-43)^ and with society/community^(41)^ Support and interaction between spouses and society^(38,41,42,45,46)^ Collaborative care between the couple^(38,39,45,46)^ Capacity to resist sexual changes^(41)^ Development as a couple^(39,41,45)^ Feeling of unity^(41,46)^ Becoming closer^(39,45)^ Coping with and repairing self-image^(38,41,46)^ Coping with the impact of ostomy and cancer treatment on sexual relationships^(45)^ Reshaping identity after ostomy^(41)^ Preservation of the identity of the “I” by the other^(41)^ Sexual repertoire celebration and expansion^(41)^ Strengthening emotional and spiritual bonds at the expense of sexual intimacy^(38)^ Spiritual healing^(38)^ Rediscovering personal value and the meaning of life^(38)^
	**Negatives**	Limitations in daily activities, in marital relations^(38, 41, 43, 44)^ and in sexual life^(38,39,42-44)^ Changes in physical and rest needs^(42-44)^ Changes in sexual style and pattern^(39,42,44,46)^ Relationship strain^(38,42,46)^ Social isolation^(38,43,44,46)^ Low satisfaction^(38,39,44)^ and low self-esteem^(42,44,46)^ Change of identity^(38)^ Significant emotional distress^(41)^

Regarding the nature of the experience, the studies analyzed describe a set of challenges and transformations experienced by couples. It was reported that this experience constitutes a constant challenge, marked by the simultaneous coexistence with both recent and past physical and psychosocial concerns^(41,46)^. Feelings of disconnection between spouses were identified, expressed by the perception of “being in the same space but not connected”^(45)^, as well as a fluctuation between individuality (“I”) and relational identity (“we”)^(45)^. There are also reports of processes of differentiation and affirmation of the conjugal “we”^(45)^, as well as the inevitable occurrence of periods of isolation between the members of a couple^(45)^.

Among the factors that facilitated the adaptation process, interdisciplinary support^(38,39,40,42)^ and pre- and post-surgical counseling stood out^(39,40,42)^. The provision of collaborative care^(38,39,45,46)^ and the redistribution of responsibilities within the couple^(45,46)^ were equally relevant. Spousal support^(39,41,42,46)^ and supportive care provided by health professionals^(41)^ emerged as determinants, based on self-care capacity^(40,42)^, marital communication^(39,41)^, and community interaction^(41)^. Other elements considered facilitating included thinking in global terms^(41)^, setting shared goals and mutual encouragement^(38,46)^, the availability of information and support from healthcare professionals^(44)^, as well as a positive attitude towards life^(40,46)^, the hope of resuming a normal life after ostomy^(43,46)^, mutual trust^(38)^, empathy and interpersonal understanding^(45)^, the use of a sense of “we” to overcome adversity^(45)^, non-sexual touch^(38)^, contact with nature^(38)^, a stable socioeconomic condition^(41)^, and faith^(38)^.

On the other hand, negative feelings associated with ostomy were highlighted as inhibiting factors, such as fear^(39,43,44,46)^, insecurity^(43,44,46)^, anxiety^(38,43,46)^, depression^(38)^, shame^(38,43)^, worry^(38,40,44)^, and psychological stress^(38,42)^. Lack of information and health education^(38,40,41,42,46)^, decreased libido^(38,39,42,43,46)^, perceived decrease in attractiveness^(38,42,44,46)^, lack of communication and support from health professionals^(39,42)^, lack of demonstrated interest from the spouse^(38)^, and financial difficulties^(43,44)^ were also identified as relevant barriers to the adaptation process.

The analysis of studies also allows us to identify a set of both positive and negative results. Concerning positive results, acceptance of the new living conditions^(38,40,42,45,46)^, improved communication between the couple^(38,39,41,42,43)^ and with the community^(41)^, strengthened marital and social support^(38,42,43,45,46)^, and the implementation of collaborative care within the relationship^(38,39,45,46)^ were reported. There was also evidence of resilience to changes in sexual life^(41)^, development as a couple^(39,41,45)^, a strengthening of the feeling of unity^(41,46)^, and a perception of greater closeness between partners^(39,45)^. Coping and repairing processes of self-image^(38,41,46)^, overcoming the impacts of ostomy and cancer treatment on sexuality^(45)^, reformulating identity after ostomy^(41)^, and preserving the identity of the “I” through the other^(41)^ were also identified. Additionally, experiences of celebrating and expanding the sexual repertoire^(41)^, strengthening emotional and spiritual bonds at the expense of sexual intimacy^(38)^, spiritual healing^(38)^, and rediscovering personal value and the meaning of life were reported^(38)^.

Finally, regarding the negative outcomes, limitations in activities of daily living, marital relationships^(39,41,43,44)^ and sexual life^(38,39,42,43,44)^, changes in physical and rest needs^(42,43,44)^, as well as modifications in sexual style and pattern were reported ^(39,42,44,46)^. There were also indications of relational strain^(38,42,46)^, social isolation^(38,43,44,46)^, low marital satisfaction^(38,39,44)^, low self-esteem^(42,44,46)^, identity changes^(38)^, and significant emotional distress^(41)^.

Furthermore, the gaps identified in the evidence refer to the scarcity of primary studies that: i) focus on couples’ experiences facing a BEO due to CRC; ii) include the marital dyad as participants; iii) describe the dynamics of these couples’ experience, namely the change processes they undergo; and iv) address couples’ experiences as a process of multiple transitions—health/illness and situational—experienced by the couple.

## DISCUSSION

The results reflect the existence of a limited number of studies on the experience of couples facing a BEO due to CRC, with particular emphasis on studies focusing on couples’ sexuality (five of the nine studies)^(38,39,40,41,42)^, privileging, in most cases, the individual perspective. This scarcity of studies indicates that this phenomenon is still understudied, pointing to the need for primary studies to be developed. In the evidence accessed, only two studies had the couple as direct participants^(41,45)^, with the remaining studies accessing data on couples’ experiences through an ostomized person^(38,39,40,43,44)^ or the spouse^(46)^. This methodological option may compromise the understanding of marital experience of BEO due to CRC, since relational phenomena are, by nature, interdependent. As Revenson *et al.* ^(47)^ point out, phenomena with high functional and symbolic impact, such as an ostomy resulting from CRC, should be understood as shared experiences, which requires methodological approaches capable of capturing the reciprocity of meanings, emotions, and coping strategies within the marital dyad. In this regard, several authors^(48,49,50)^ emphasize that the use of dyadic data allows for more reliable access to the complexity of the marital relationship. When only one member of the couple is included in the study, the data obtained may reflect partial perceptions, biased by emotional factors, narrative limitations, or difficulties in expressing relational experiences comprehensively.

The analysis of the objectives of the studies included in this scoping review revealed that, in general, they focus on exploring couples’ experiences in the face of the impact of ostomy associated with CRC, with particular emphasis on couples’ sexuality, quality of life, and challenges related to adaptation and adjustment to the new condition. The included studies acknowledge several changes experienced by couples living with BEO due to CRC, particularly in the physical, emotional, relational, social, and spiritual dimensions.

The studies included in this scoping review use the concepts of adaptation and adjustment when referring to experience. Although frequently used, these concepts are rarely accompanied by definitions, which hinders their understanding, analytical distinction, and comparison between studies. Nevertheless, based on the analysis carried out, it is understood that adaptation is multidimensional^(38,41,42,45)^ and involves responding to and integrating into new circumstances. It is essential for maintaining couples’ emotional, physical, and psychological well-being and marital relationship quality^(38,41,42,45)^. Adjustment, on the other hand, corresponds to a continuous trajectory of practical response to the physical, psychological, and social changes imposed by the condition^(39,43,45)^. In general, adjustment is described as strategies adopted to deal with sexual disruptions after surgery^(39)^, the modifications made to daily routines, personal care and social interactions^(43)^, and, from a relational perspective, implies a delicate balance between preserving the individual identity of each element and maintaining the affective and functional bond of the dyad^(45)^. Evidence available shows that these concepts are interconnected. In Tripaldi’s study^(42)^, the concepts of adaptation and adjustment are implicitly present, but are not presented independently or conceptually differentiated, appearing integrated in the discussion of coping strategies, changes in identity and relational dynamics. However, a more detailed analysis reveals that adaptation is conceived as a response — encompassing integration and acceptance^(40,43)^ — while adjustment reflects an active and dynamic process of change and negotiation^(45)^.

It is also important to highlight that none of the studies explicitly used the concept of transition to describe the experience lived by couples facing a BEO in the context of CRC. Since transition is defined as a process and result of passing from one condition or state to another, particularly those related to health and role performance^(24,25)^, it would be expected that this concept would be used in research on the experience of these couples. This absence highlights a significant weakness in the theoretical approach to the problem under study and suggests the need to deepen the understanding of this experience, in light of the perspective of Transition Theory, thus contributing to a more comprehensive and sensitive analysis of the procedural dynamics involved.

In relation to the nature of the experience, studies highlight the challenges and changes experienced by couples. Several authors^(41,46)^ state that this experience constitutes a constant challenge, since couples simultaneously deal with emerging concerns related to the disease and the ostomy, and with previously existing psychosocial issues, which may be reactivated or increased in the context of adversity. However, they reveal that the experience is lived differently among the couples studied, oscillating between feelings of marital disconnection^(45)^ and strengthening of the bond^(39,45)^, between the “I” and the “we”, revealing the complexity of adapting to a new identity, conditioned by the ostomy and the repercussions of the disease.

Scientific evidence demonstrates that this alternation between disconnection and strengthening of a marital relationship^(21)^, in the context of an ostomy, does not occur randomly, but results from the complex interaction between multiple relational factors. Hence, effective communication plays a decisive role: couples who manage to share emotions, negotiate roles and exchange information openly tend to reinforce the feeling of belonging and unity (“we”)^(51)^.These authors identify that dyadic effectiveness, i.e., shared confidence in the ability to cope with cancer, is facilitated by fluid communication, mutual interest in information, and common coping strategies, while barriers to this communication result in less cohesion and greater emotional distress.

Alignment of expectations and perceptions between spouses is also fundamental. Qin *et al.* ^(52)^ emphasize that congruent approaches in the perception of stress and adaptive responses, namely between fear of relapse and joint approach, contribute to higher levels of resilience and marital adjustment. When perceptions diverge — for instance, when an ostomized patient minimizes the impact while the spouse feels overwhelmed — a growing sense of disconnection and maladjustment tends to arise in the dyad.

Additionally, social support and access to professional resources, such as coping programs for couples with CRC, have been shown to positively impact the shared experience. Structured interventions that promote stress communication and community coping, as well as educational strategies designed to involve both partners, have been associated with improvements in relationship satisfaction and individual mental health^(51,53,54)^. In this vein, and according to the data obtained in this review, it appears that factors such as good communication, shared responsibility, emotional alignment, and adequate professional support favor the emergence of a renewed sense of “we”. This shared sense allows couples to face ostomy and the repercussions of CRC with greater cohesion and mutual adjustment. Conversely, inhibiting factors point to barriers to adaptation, namely feelings of shame, fear, anxiety and depression, lack of information, professional support, and economic difficulties.

The results of the review, by identifying support from healthcare professionals as a facilitating factor in the experience and a lack of information and health education, as well as the absence of supportive professional communication, as inhibiting factors, seem to highlight the need for professionals—particularly nurses—to deepen their knowledge of this phenomenon in order to provide a basis for interventions that effectively support couples facing this experience.

A critical reading of results reveals that couples’ experiences with an BEO due to CRC is simultaneously transformative and vulnerable, integrating positive dimensions such as acceptance, mutual support, collaboration in care, and reinforcement of marital identity, and negative dimensions such as changes in sexual intimacy, emotional isolation, and the impact on self-esteem and individual identity. This coexistence of polarities suggests that the experience of BEO due to CRC, when lived in a marital context, is neither linear nor univocal, and confirms that facing an ostomy due to CRC goes beyond the limits of individual adaptation, constituting a relational experience that involves the renegotiation of roles and meanings within the marital dyad. This perspective finds support in recent literature. Lin *et al.* ^(55)^, for instance, emphasize that functional changes and psychological disturbances significantly interfere with marital intimacy and the perception of personal value, reinforcing the importance of interventions oriented towards the dyadic relationship. In this regard, the data from this review show that the positive and negative outcomes of the marital experience coexist and interact, supporting the need for specific intervention strategies centered on the couple, such as sexual and marital counseling, educational programs for couples, and ongoing emotional support, which favor processes of mutual adjustment^(53,54)^.

### Limitations

The limitations of this scoping review comprise the inclusion of a small number of studies addressing the experience of couples facing BEO due to CRC as a unit. Although the evidence favors couples’ experiences, the fact that most of the selected studies included only one ostomized member of the couple may have generated some bias in the perception of the results of those studies and, therefore, also in those of this review. Additionally, there is a methodological limitation related to the impossibility of accessing the full text of one of the articles identified during the selection process. Despite efforts, including consulting institutional repositories, academic libraries, and contacting the authors directly, access could not be guaranteed, which restricted the body of evidence available for analysis.

### Contributions to Nursing

Although the nature of this review does not allow for direct practical implications, the results point to important elements that can guide healthcare professionals, specifically nurses. The results of this scoping review highlight the demands and transformations that couples may face in this situation, emphasizing the importance of ensuring continuous support and health education through interventions that facilitate the processes of change faced by the couple, promoting, among other outcomes, resilience and marital well-being.

## CONCLUSION

This review, without a time limit, concludes that there is a scarcity of scientific evidence on the phenomenon. Available evidence originated from six countries. Although reporting on couples’ experiences with a BEO due to CRC, participants were mostly a person with an ostomy. The concepts of adaptation and adjustment were prominent in describing that experience. The experience of these couples was marked by the need for continuous and mutual adjustments to the changes imposed by the ostomy and by a duality between challenges and opportunities for growth. This experience was characterized in three categories: i) the nature of the experience; ii) facilitating and inhibiting factors; and iii) positive and negative outcomes.

Focusing on the construction of knowledge relevant to nursing, the development of primary research that improves the understanding of the experience of couples facing BEO due to CRC is considered important. In this context, the study of the dynamics underlying their adaptation, adjustment, or transition in the face of such conditions is highlighted as relevant, i.e., the study of the change process(es) faced by the dyad. The findings of these studies may constitute a knowledge base for the subsequent development of interventions that facilitate these processes and achieve health outcomes for these couples.

## Data Availability

The entire dataset supporting the results of this study is available upon request to the corresponding author.
