# Supplementary material for: Couples’ experiences with ostomy due to colorectal cancer in one of the partners: a scoping review
Source: Rev Esc Enferm USP. 2026 Feb 23;60:e20250250. doi: 10.1590/1980-220X-REEUSP-2025-0250en (PMC12945306; doi:10.1590/1980-220X-REEUSP-2025-0250en)
Supplement: Translation

# Translation: Experiência do casal em caso de ostomia por cancro colorretal de um dos membros: *scoping review*

**DOI:** 10.1590/1980-220X-REEUSP-2025-0250pt

**Authors:** Ricardo Emanuel Sousa Mestre, Ana Beatriz Gomes Dias, Célia Maria Gonçalves Simão de Oliveira, Patrícia Vinheiras Alves

**Affiliations:** 1 Escola Superior de Saúde Atlântica, Lisboa, Portugal.; 2 Instituto Português de Oncologia de Lisboa Francisco Gentil, Lisboa, Portugal.; 3 Universidade de Lisboa, Escola Superior de Enfermagem, Lisboa, Portugal.; 4 Universidade de Lisboa, Escola Superior de Enfermagem, Centro de Investigação Inovação e Desenvolvimento em Enfermagem de Lisboa, Lisboa, Portugal.

**Keywords:** Adaptação Psicológica, Ajustamento Social, Neoplasias Colorretais, Características da Família, Estomia

## Abstract

**Objetivo:**

Mapear a evidência científica sobre a experiência conjunta do casal que enfrenta uma ostomia de eliminação intestinal por cancro colorretal.

**Método:**

Realizou-se uma *scoping review*, conduzida de acordo com as diretrizes do JBI. A pesquisa foi efetuada em 13 bases de dados e repositórios, com critérios de inclusão definidos pela abordagem PCC (Participantes, Conceito e Contexto).

**Resultados:**

Foram incluídos nove estudos, que permitiram mapear a experiência do casal e resumir as suas características segundo a natureza, os fatores influenciadores e os resultados decorrentes dessa experiência; na nomeação desta, predominaram os conceitos de adaptação e/ou de ajustamento.

**Conclusão:**

A experiência do casal com ostomia por cancro colorretal é vivida de forma divergente entre os casais, envolvendo desafios físicos, emocionais e relacionais. Identificam-se lacunas na evidência científica, constatando-se a necessidade de investigação primária que possibilite a compreensão sobre esta experiência, nomeadamente a dinâmica ou o processo experienciado por esses casais.

## INTRODUÇÃO

A nível mundial, o cancro colorretal (CCR) foi o terceiro tipo de cancro mais diagnosticado em 2020, ocupando o terceiro lugar em termos de incidência e o segundo em termos de mortalidade^(1)^. Apesar da gravidade, verificou-se, nos últimos anos, uma redução na taxa de mortalidade por CCR atribuída aos avanços no diagnóstico precoce e no tratamento^(2)^. A cirurgia constitui o tratamento de eleição^(3,4,5)^, frequentemente resultando na construção de uma ostomia de eliminação intestinal (OEI) provisória ou definitiva^(6)^. Embora não existam dados globais precisos, estima-se que cerca de 1 milhão de pessoas nos Estados Unidos^(7,8)^, 1 milhão na China e 700 mil na Europa vivam atualmente com pelo menos uma ostomia^(7)^, sendo expectável que este número continue a aumentar em consequência do crescimento da incidência do CCR^(9)^.

O diagnóstico e tratamento do CCR representam uma experiência profundamente desafiante, marcada por sofrimento físico e emocional, tristeza, ansiedade, medo, e insegurança^(10)^. A presença de uma OEI agrava as alterações vivenciadas pela pessoa, gerando desafios adicionais relacionados à imagem corporal, autoestima, sexualidade, e participação em atividades sociais, físicas e de lazer^(11,12)^. Essas mudanças afetam diretamente a vida quotidiana e perceção da qualidade de vida^(11,12,13)^.

Partindo do princípio de que os eventos vivenciados por um membro da família afetam os restantes^(14)^, é plausível afirmar que, no contexto de uma relação conjugal, essas mudanças são experienciadas pelo casal como unidade. Considera-se casal a união de duas pessoas adultas que mantêm uma relação significativa, íntima e duradoura^(15)^, partilhando um projeto de vida comum^(16)^. Neste contexto, o cônjuge assume frequentemente o papel de cuidador principal, enfrentando também mudanças substanciais no seu quotidiano, com repercussões emocionais, sociais e relacionais^(17,18)^. Assim, entende-se que a experiência vivenciada pelo casal em resposta ao CCR e à OEI reflete-se em reajustes conjuntos, com impacto significativo na relação conjugal, bem como na saúde mental e física de ambos os cônjuges^(18,19,20)^.

A experiência do casal que enfrenta um desafio dessa natureza é reconhecida como um processo dinâmico e evolutivo, caracterizado por transições contínuas^(20)^, adaptações mútuas^(20,21,22)^ e ajustamentos conjugais^(20,23)^. Essa perspetiva sublinha que a experiência do casal está em constante transformação, refletindo-se na forma como os cônjuges se adaptam e reorganizam diante das mudanças impostas pela condição de saúde.

Nesse contexto, as mudanças provocadas pelo CCR e pela OEI desencadeiam um processo que pode ser conceptualizado como transição, entendido como a passagem de uma condição ou estado para outro^(24,25)^. A transição, enquanto experiência humana e dinâmica, exige que a pessoa ou, no caso presente, o casal se envolva em processos de adaptação e ajustamento^(24,25,26)^. Esse processo é complexo e ocorre ao longo do ciclo vital, sendo desencadeado por eventos críticos – designadamente os relacionados à saúde e ao desempenho de papéis -, e resultando em mudanças de papéis, aquisição de novos conhecimentos, mudanças naqueles papéis e adaptação de comportamentos^(25,27)^.

Assim, a adaptação e o ajustamento emergem como elementos centrais àquele processo, influenciando a capacidade do casal para reorganizar a sua vida diante da nova condição^(27,28)^. A adaptação se refere à capacidade de reorganizar a sua vida e identidade, integrando as novas exigências impostas pela condição de saúde, e promovendo o bem-estar, a continuidade da relação conjugal e a relação com o mundo exterior^(29)^. O ajustamento, por sua vez, diz respeito ao envolvimento ativo do casal, sendo condicionado pelo conhecimento que ambos possuem sobre a sua própria situação e pela capacidade de gerir as mudanças e integrar a nova condição de vida^(27,30)^. Desse modo, a adaptação é traduzida na integração das mudanças na vida quotidiana, enquanto o ajustamento reflete o processo contínuo de gerir e lidar com essas mudanças.

O casal que enfrenta uma OEI por CCR constitui um grupo particular no que diz respeito às necessidades de cuidados de saúde^(17)^. No entanto, a evidência científica tem se centrado predominantemente na experiência individual da pessoa com OEI, do cônjuge enquanto cuidador ou da família em geral, negligenciando a compreensão da experiência partilhada do casal como unidade. Essa lacuna é particularmente relevante, uma vez que a vivência da OEI por CCR não se limita ao indivíduo afetado, mas abrange o casal, influenciando a sua relação, comunicação, papéis e bem-estar emocional. A enfermagem, enquanto disciplina que privilegia o cuidado centrado na pessoa, tem a responsabilidade de compreender esta experiência partilhada, promovendo intervenções que respondam às necessidades do casal como uma unidade^(27,31,32)^. Uma pesquisa preliminar nas bases de dados *JBI Evidence Synthesis*, Cochrane, CINAHL, MEDLINE, PROSPERO, *Open Science Framework* e *Figshare*, utilizando os termos de pesquisa “*transition*”, “*adaptation*”, “*adjustment*”, “*colorectal neoplasms*”, “*ostomy*” e “*couple*”, nos idiomas português, inglês, francês e espanhol, não identificou qualquer revisão da literatura, publicada ou em curso, que tenha como foco de análise o casal enquanto unidade participante no processo de vivência de uma OEI por CCR.

Neste sentido, justifica-se a realização desta *scoping review*, que tem como objetivo mapear^(33,34,35)^ a evidência científica sobre a experiência conjunta do casal que enfrenta uma OEI por CCR, identificando a evidência científica disponível e as lacunas nesse conhecimento, tendo como finalidade fundamentar futura investigação que contribua para o desenvolvimento do conhecimento em enfermagem relativamente a este fenómeno nesta população.

## MÉTODO

### Tipo de Estudo

Trata-se de uma scoping review realizada segundo a metodologia proposta pelo *JBI* ^(33,34)^ e as diretrizes do *Preferred Reporting Items for Systematic Reviews and Meta-Analyses extension for Scoping Reviews* (PRISMA-ScR) *checklist* ^(34,36)^. O protocolo foi previamente registado na plataforma *Open Science Framework*, com o DOI: 10.17605/OSF.IO/E8WJD. Durante o desenvolvimento da revisão, constatou-se ser necessário introduzir alterações ao protocolo: foi reformulada a terminologia usada no título, objetivo e questões de revisão, devido à constatação de que os artigos selecionados não utilizavam o conceito de transição para nomear a experiência dos casais.

Foi definida como questão de revisão “Qual é a evidência científica disponível sobre a experiência conjunta do casal que enfrenta uma OEI por CCR?”, bem como as subquestões “Quais são os estudos existentes sobre a experiência do casal perante uma OEI por CCR?”, “Quais são as características da população na evidência disponível sobre esta experiência?”, “Que conceitos são utilizados para descrever a experiência do casal?” e “Como se caracteriza, na evidência disponível, a experiência do casal?”.

### Critérios de Seleção

Os critérios de elegibilidade foram definidos com base na mnemónica PCC (Participantes, Conceito e Contexto), conforme descrito por Peters *et al.* ^(33,34)^. No que concerne aos participantes, a revisão considerou estudos que incluíssem adultos (idade ≥ 19 anos) em uma relação conjugal, em que um dos elementos fosse ser portador de OEI devido ao CCR, em que os dados tenham sido colhidos junto ao casal, à pessoa com ostomia ou ao cônjuge. Para efeitos de elegibilidade, considerou-se como “casal” qualquer referência explícita nos estudos a termos como “casal”, “companheiro(a)”, “cônjuge”, “parceiro(a)” ou “esposo(a)”, independentemente do género ou estado civil. Excluíram-se estudos em que a OEI não tivesse sido construída na sequência de CCR.

Relativamente ao conceito, foram incluídos estudos que abordassem a experiência do casal diante da OEI resultante de CCR, bem como estudos que investigassem a transição, a adaptação ou o ajustamento do casal perante essa condição. Foram excluídos os estudos que não enfocassem a experiência, a transição, o ajustamento ou a adaptação do casal, bem como aqueles que exploravam esses conceitos apenas a nível individual, centrando-se exclusivamente na pessoa com ostomia ou no cônjuge. Quanto ao contexto, não foram impostas restrições relacionadas a fatores culturais, étnicos, de género ou de localização geográfica.

A revisão incluiu estudos primários, secundários e uma tese. Nos estudos selecionados, foi identificada revisão da literatura. Contudo, não se verificou qualquer duplicação de dados decorrente da inclusão de estudos primários nesta revisão, não havendo, assim, necessidade de excluir os referidos estudos primários^(34)^. Devido à finalidade da revisão, excluíram-se livros, capítulos de livros e artigos de opinião. Foram considerados estudos publicados em inglês, português, francês e espanhol, não tendo sido aplicada qualquer restrição temporal.

### Colheita de Dados

A pesquisa inicial foi realizada entre maio e junho de 2024, e atualizada em julho de 2025, seguindo uma abordagem multifásica, com o objetivo de identificar o maior número possível de estudos e publicações relevantes. Na primeira fase, realizou-se pesquisa nas bases de dados eletrónicas MEDLINE (via EBSCO), CINAHL (via EBSCO), SciELO, *Cochrane Database of Systematic Reviews* (via EBSCO), *JBI Evidence Synthesis, Psychology and Behavioral Sciences Collection* (via EBSCO), PsycINFO (via EBSCO), *Web of Science* e Scopus. Na segunda fase, com o objetivo de integrar literatura cinzenta, efetuaram-se pesquisas no *Open Access Theses and Dissertations*, *ProQuest Dissertation and Theses Database*, Repositório Científico de Acesso Aberto de Portugal (RCAAP) e *Google Scholar*. Não foi possível incluir dados provenientes do *OpenGrey* e do *DART-Europe*, uma vez que se encontravam inativos à data da recolha de informação. Por fim, na terceira fase, procedeu-se à análise das referências bibliográficas dos estudos incluídos, visando identificar artigos adicionais potencialmente relevantes para o estudo.

Os termos do *Medical Subject Headings* identificados previamente foram incluídos e adaptados para cada fonte de informação utilizada, desenvolvendo-se uma estratégia de pesquisa completa com operadores de truncagem e booleanos apropriados (Quadro 1).

**Quadro 1:** Estratégia de pesquisa – Lisboa, Portugal, 2025.

Base de dados	Estratégia de pesquisa
CINAHL *complete* (EBSCO)	(XB (caregiv* OR “care partner” OR conjugal OR coupl* OR dyad* OR “family member” OR husband* OR “life partner” OR marital* OR married* OR mutuality OR partner* OR spous* OR wife OR wive) OR MH (caregivers OR marriage OR “married women” OR “married men” OR spouses OR “significant other”)) AND (XB (stoma* OR enterostomy OR ostomy OR ileostomy OR colostomy) OR MH ostomy+) AND (XB (“colorectal cancer” OR “CRC” OR “bowel cancer” OR “colon cancer” OR “rectal cancer”) OR MH (“cancer patients” OR “intestinal neoplasm+” OR neoplasms)) AND (XB (Change* OR experience* OR “life-change event” OR “life changing event” OR “life-changing event” OR “life change*” OR transition* OR adjustment* OR adaptation) OR MH (“life change events+” OR “adaptation, psychological+” OR “life experiences+” OR “social adjustment”))
MEDLINE *Complete* (EBSCO)	(XB (caregiv* OR “care partner” OR conjugal OR coupl* OR dyad* OR “family member” OR husband* OR “life partner” OR marital* OR married* OR mutuality OR partner* OR spous* OR wife OR wive) OR MH (caregivers OR marriage OR spouses)) AND (XB (stoma* OR enterostomy OR ostomy OR ileostomy OR colostomy) OR MH (ostomy+ OR “surgical stomas”)) AND (XB (“colorectal cancer” OR “CRC” OR “bowel cancer” OR “colon cancer” OR “rectal cancer”) OR MH (“intestinal neoplasms+” OR neoplasms)) AND (XB (Change* OR experience* OR “life-change event” OR “life changing event” OR “life-changing event” OR “life change*” OR transition* OR adjustment* OR adaptation) OR MH (“life change events” OR “adaptation, psychological+” OR “social adjustment”))
*Psychology & Behavioral Sciences Collection* (EBSCO)	(XB (caregiv* OR “care partner” OR conjugal OR coupl* OR dyad* OR “family member” OR husband* OR “life partner” OR marital* OR married* OR mutuality OR partner* OR spous* OR wife OR wive) OR SU (caregiv* OR “care partner” OR conjugal OR coupl* OR dyad* OR “family member” OR husband* OR “life partner” OR marital* OR married* OR mutuality OR partner* OR spous* OR wife OR wive)) AND (XB (stoma* OR enterostomy OR ostomy OR ileostomy OR colostomy) OR SU (stoma* OR enterostomy OR ostomy OR ileostomy OR colostomy)) AND (XB (“colorectal cancer” OR “CRC” OR “bowel cancer” OR “colon cancer” OR “rectal cancer”) OR SU (“colorectal cancer” OR “CRC” OR “bowel cancer” OR “colon cancer” OR “rectal cancer”)) AND (XB (Change* OR experience* OR “life-change event” OR “life changing event” OR “life-changing event” OR “life change*” OR transition* OR adjustment* OR adaptation) OR SU (Change* OR experience* OR “life-change event” OR “life changing event” OR “life-changing event” OR “life change*” OR transition* OR adjustment* OR adaptation))
PsycInfo (APA PsycNet)	(title: (caregiv*) OR title: (“care partner”) OR title: (conjugal) OR title: (coupl*) OR title: (dyad*) OR title: (“family member”) OR title: (husband*) OR title: (“life partner”) OR title: (marital*) OR title: (married*) OR title: (mutuality) OR title: (partner*) OR title: (spous*) OR title: (wife) OR title: (wive)) OR (abstract: (caregiv*) OR abstract: (“care partner”) OR abstract: (conjugal) OR abstract: (coupl*) OR abstract: (dyad*) OR abstract: (“family member”) OR abstract: (husband*) OR abstract: (“life partner”) OR abstract: (marital*) OR abstract: (married*) OR abstract: (mutuality) OR abstract: (partner*) OR abstract: (spous*) OR abstract: (wife) OR abstract: (wive)) OR (MeSH: (Caregivers) OR MeSH: (marriage) OR MeSH: (spouses))) AND ((title: (caregiv*) OR title: (“care partner”) OR title: (conjugal) OR title: (coupl*) OR title: (dyad*) OR title: (“family member”) OR title: (husband*) OR title: (“life partner”) OR title: (marital*) OR title: (married*) OR title: (mutuality) OR title: (partner*) OR title: (spous*) OR title: (wife) OR title: (wive)) OR (abstract: (caregiv*) OR abstract: (“care partner”) OR abstract: (conjugal) OR abstract: (coupl*) OR abstract: (dyad*) OR abstract: (“family member”) OR abstract: (husband*) OR abstract: (“life partner”) OR abstract: (marital*) OR abstract: (married*) OR abstract: (mutuality) OR abstract: (partner*) OR abstract: (spous*) OR abstract: (wife) OR abstract: (wive)) OR (MeSH: (Caregivers) OR MeSH: (marriage) OR MeSH: (spouses)) AND (title: (stoma* OR enterostomy OR ostomy OR ileostomy OR colostomy)) OR (abstract: (stoma* OR enterostomy OR ostomy OR ileostomy OR colostomy)) OR (MeSH: (Ostomy) OR MeSH: (“surgical stomas”)) AND (title: (“colorectal cancer”) OR title: (“CRC”) OR title: (“bowel cancer”) OR title: (“colon cancer”) OR title: (“rectal cancer”)) OR (abstract: (“colorectal cancer”) OR abstract: (“CRC”) OR abstract: (“bowel cancer”) OR abstract: (“colon cancer”) OR abstract: (“rectal cancer”)) OR (MeSH: (“intestinal neoplasms”) OR MeSH: (neoplasms)) AND (title: (Change*) OR title: (experience*) OR title: (“life-change event”) OR title: (“life changing event”) OR title: (“life-changing event”) OR title: (“life change*”) OR title: (transition*) OR title: (adjustment*) OR title: (adaptation)) OR (abstract: (Change*) OR abstract: (experience*) OR abstract: (“life-change event”) OR abstract: (“life changing event”) OR abstract: (“life-changing event”) OR abstract: (“life change*”) OR abstract: (transition*) OR abstract: (adjustment*) OR abstract: (adaptation)) OR (MeSH: (“life change events”) OR MeSH: (“adaptation, psychological”) OR MeSH: (“social adjustment”))
Scopus	(TITLE-ABS-KEY (caregiv* OR “care partner” OR conjugal OR coupl* OR dyad* OR “family member” OR husband* OR “life partner” OR marital* OR married* OR mutuality OR partner* OR spous* OR wife OR wive )) AND ( TITLE-ABS-KEY ( stoma*)) AND (TITLE-ABS-KEY ( “colorectal cancer” OR “CRC” OR “bowel cancer” OR “colon cancer” OR “rectal cancer”)) AND (TITLE-ABS-KEY (Change* OR experience* OR “life-change event” OR “life changing event” OR “life-changing event” OR “life change*” OR transition* OR adjustment* OR adaptation))
*Web of Science Core Collection* (*Web of Science*)	(TS=(caregiv* OR “care partner” OR conjugal OR coupl* OR dyad* OR “family member” OR husband* OR “life partner” OR marital* OR married* OR mutuality OR partner* OR spous* OR wife OR wive)) AND (TS=(stoma* OR enterostomy OR ostomy OR ileostomy OR colostomy)) AND (TS=(“colorectal cancer” OR “CRC” OR “bowel cancer” OR “colon cancer” OR “rectal cancer”)) AND (TS=(Change* OR experience* OR “life-change event” OR “life changing event” OR “life-changing event” OR “life change*” OR transition* OR adjustment* OR adaptation))
SciELO *Citation Index* (*Web of Science*)	(TS=(caregiv* OR “care partner” OR conjugal OR coupl* OR dyad* OR “family member” OR husband* OR “life partner” OR marital* OR married* OR mutuality OR partner* OR spous* OR wife OR wive)) AND (TS=(stoma* OR enterostomy OR ostomy OR ileostomy OR colostomy)) AND (TS=(“colorectal cancer” OR “CRC” OR “bowel cancer” OR “colon cancer” OR “rectal cancer”)) AND (TS=(Change* OR experience* OR “life-change event” OR “life changing event” OR “life-changing event” OR “life change*” OR transition* OR adjustment* OR adaptation))
Cochrane	(caregiv* OR “care partner” OR conjugal OR coupl* OR dyad* OR “family member” OR husband* OR “life partner” OR marital* OR married* OR mutuality OR partner* OR spous* OR wife OR wive):ti,ab,kw *MeSH descriptor: [Caregivers] explode all trees* *MeSH descriptor: [Marriage] explode all trees* *MeSH descriptor: [Spouses] explode all trees* *#1 OR #2 OR #3 OR #4* (stoma* OR enterostomy OR ostomy OR ileostomy OR colostomy):ti,ab,kw *MeSH descriptor: [Ostomy] explode all trees* *MeSH descriptor: [Surgical Stomas] explode all trees* *#6 OR #7 OR #8* *(“colorectal cancer” OR “CRC” OR “bowel cancer” OR “colon cancer” OR “rectal cancer”):ti,ab,kw* *MeSH descriptor: [Intestinal Neoplasms] explode all trees* *MeSH descriptor: [Neoplasms] explode all trees* *#10 OR #11 OR #12* (Change* OR experience* OR “life-change event” OR “life changing event” OR “life-changing event” OR life NEXT change* OR transition* OR adjustment* OR adaptation):ti,ab,kw *MeSH descriptor: [Life Change Events] explode all trees* *MeSH descriptor: [Adaptation, Psychological] explode all trees* *MeSH descriptor: [Social Adjustment] explode all trees* *#14 OR #15 OR #16 OR #17* *#5 AND #9 AND #13 AND #18*
*ProQuest Dissertations & Theses Citation Index* (*Web of Science*)	(TS=(caregiv* OR “care partner” OR conjugal OR coupl* OR dyad* OR “family member” OR husband* OR “life partner” OR marital* OR married* OR mutuality OR partner* OR spous* OR wife OR wive)) AND (TS=(stoma* OR enterostomy OR ostomy OR ileostomy OR colostomy)) AND (TS=(“colorectal cancer” OR “CRC” OR “bowel cancer” OR “colon cancer” OR “rectal cancer”)) AND (TS=(Change* OR experience* OR “life-change event” OR “life changing event” OR “life-changing event” OR “life change*” OR transition* OR adjustment* OR adaptation))
RCAAP (EBSCO)	(TI (caregiv* OR “care partner” OR conjugal OR coupl* OR dyad* OR “family member” OR husband* OR “life partner” OR marital* OR married* OR mutuality OR partner* OR spous* OR wife OR wive) OR TI (caregiv* OR “care partner” OR conjugal OR coupl* OR dyad* OR “family member” OR husband* OR “life partner” OR marital* OR married* OR mutuality OR partner* OR spous* OR wife OR wive) OR SU (caregiv* OR “care partner” OR conjugal OR coupl* OR dyad* OR “family member” OR husband* OR “life partner” OR marital* OR married* OR mutuality OR partner* OR spous* OR wife OR wive)) AND (TI (stoma* OR enterostomy OR ostomy OR ileostomy OR colostomy) OR AB (stoma* OR enterostomy OR ostomy OR ileostomy OR colostomy) OR SU (stoma* OR enterostomy OR ostomy OR ileostomy OR colostomy)) AND (TI (“colorectal cancer” OR “CRC” OR “bowel cancer” OR “colon cancer” OR “rectal cancer”) OR AB (“colorectal cancer” OR “CRC” OR “bowel cancer” OR “colon cancer” OR “rectal cancer”) OR SU (“colorectal cancer” OR “CRC” OR “bowel cancer” OR “colon cancer” OR “rectal cancer”)) AND (TI (Change* OR experience* OR “life-change event” OR “life changing event” OR “life-changing event” OR “life change*” OR transition* OR adjustment* OR adaptation) OR AB (Change* OR experience* OR “life-change event” OR “life changing event” OR “life-changing event” OR “life change*” OR transition* OR adjustment* OR adaptation) OR SU (Change* OR experience* OR “life-change event” OR “life changing event” OR “life-changing event” OR “life change*” OR transition* OR adjustment* OR adaptation)) *Filters: Content Provider: RCAAP*
*JBI Evidence Syntesis*	stoma* OR enterostomy OR ostomy OR ileostomy OR colostomy
*Google Scholar*	*allintitle: stoma cancer experience spouse* *allintitle: stoma cancer transition spouse* *allintitle: stoma cancer adaptation spouse* *allintitle: stoma cancer adjustment spouse* *allintitle: ostomy cancer experience spouse* *allintitle: ostomy cancer transition spouse* *allintitle: ostomy cancer adaptation spouse* *allintitle: ostomy cancer adjustment spouse* *allintitle: ostomia cancro experiência casal* *allintitle: ostomia cancro transição casal* *allintitle: ostomia cancro adaptação casal* *allintitle: ostomia cancro ajustamento casal*
*Open Access Thesis and Dissertations*	abstract (caregiv* OR partner OR conjugal OR coupl* OR dyad* OR family OR husband* OR marital* OR married* OR spous* OR wife OR wive) AND abstract (stoma* OR enterostomy OR ostomy OR ileostomy OR colostomy) AND abstract (cancer* OR neoplasm*) AND abstract (Change* OR experience* OR transition* OR adjustment* OR adaptation) subject (caregiv* OR partner OR conjugal OR coupl* OR dyad* OR family OR husband* OR marital* OR married* OR spous* OR wife OR wive) AND subject (stoma* OR enterostomy OR ostomy OR ileostomy OR colostomy) AND subject (cancer* OR neoplasm*) AND subject (Change* OR experience* OR transition* OR adjustment* OR adaptation) title (caregiv* OR partner OR conjugal OR coupl* OR dyad* OR family OR husband* OR marital* OR married* OR spous* OR wife OR wive) AND title (stoma* OR enterostomy OR ostomy OR ileostomy OR colostomy) AND title (cancer* OR neoplasm*) AND title (Change* OR experience* OR transition* OR adjustment* OR adaptation)

As referências identificadas foram compiladas e carregadas na plataforma Rayyan®, tendo os registos duplicados sido removidos, o que resultou em um total de 733 documentos. A análise dos títulos e resumos dos documentos foi realizada por dois revisores independentes utilizando o método *blind-one*. As divergências foram resolvidas por um terceiro revisor^(33,36)^.

Os documentos que cumpriram os critérios de inclusão pela análise de título e resumo foram recuperados na íntegra para análise detalhada, tendo o texto completo sido lido de forma independente pelos revisores. O processo de seleção dos documentos está representado no diagrama de fluxo PRISMA-ScR (Figura 1), que ilustra, de forma sistemática, as etapas do processo^(37)^, culminando na seleção de nove documentos.

**Figura 1:** Diagrama de fluxo do Preferred Reporting Items for Systematic Reviews and Meta-Analyses extension for Scoping Reviews^(37)^.

### Análise e Tratamento de Dados

A extração e apresentação dos dados extraídos dos estudos incluídos seguiram uma estrutura especificamente delineada para esta *scoping review*, em função do objetivo e questões de revisão^(33,34)^. A extração dos dados foi realizada por dois revisores independentes, e as divergências foram resolvidas com recurso a um terceiro revisor. Foram extraídos os dados que se relacionavam ao objetivo e às questões de revisão da revisão (Quadro 2), como título, autor(es), ano, país, objetivo, metodologia e métodos do estudo, população-alvo, e principais resultados. Os dados recolhidos foram analisados através de contagem de frequência, para caracterização dos documentos incluídos e de análise de conteúdo qualitativa descritiva, para resumir as características da experiência do casal. Os resultados são apresentados em quadro e por meio de descrição narrativa.

**Quadro 2:** Apresentação dos dados extraídos – Lisboa, Portugal, 2025.

	Autor(es)/ano/país	Metodologia e métodos de estudo	População	Objetivo	Principais resultados
**Estudo 1**	Du, Chaiviboontha & Sumdaengrit/2024/China^(38)^	Paradigma qualitativo Fenomenologia Entrevista	16 pessoas com ostomia (idade entre 18 e 59 anos)	Explorar as experiências de sobreviventes de cancro colorretal, na intimidade conjugal, após a realização de uma ostomia.	O estudo identificou cinco dimensões fundamentais na experiência conjugal de sobreviventes de cancro colorretal com ostomia: intimidade física, psicológica, social, espiritual e operacional. A presença do estoma afeta negativamente o envolvimento sexual, gerando preocupações quanto à atratividade e autoestima, refletindo-se na diminuição da libido e em dificuldades de expressão afetiva. Ainda assim, as conexões emocionais e espirituais são percepcionadas como particularmente significativas, contribuindo para redefinição do sentido de identidade e de autovalor. A gestão diária (intimidade operacional) emerge como elemento crítico, exigindo colaboração, suporte do cuidador e competências de resolução de conflitos contribuindo para uma adaptação bem-sucedida.
**Estudo 2**	Afiyanti, Fitch, Helen, Andjarwati, Rudi, Prawesti & Juliastuti/2023/Indonésia^(39)^	Paradigma qualitativo Fenomenologia Entrevista	12 pessoas com ostomia (idade entre 31 e 58 anos)	Explorar a perspetiva das pessoas que vivem com ostomia acerca das suas experiências sexuais e as influências físicas, psicológicas e culturais.	O estudo evidenciou perturbações sexuais significativas em sobreviventes de cancro colorretal com ostomia, incluindo diminuição do desejo, desconforto e insatisfação, levando alguns a interromper totalmente a intimidade sexual. O apoio do cônjuge surgiu como fator positivo, potenciando melhor comunicação e maior disponibilidade para o convívio, reconhecida como benéfica pelos participantes. Muitas estratégias de adaptação se centraram no bem-estar conjugal, por vezes em detrimento das necessidades sexuais individuais. Contudo, constatou-se uma lacuna na comunicação sobre questões sexuais com profissionais de saúde. Os resultados sublinham a necessidade de apoio interdisciplinar e de aconselhamento pré e pós-cirúrgico para melhorar a saúde sexual e o relacionamento conjugal.
**Estudo 3**	Du, Wang, Du, Zou & Jin/2021/China^(40)^	Paradigma quantitativo Estudo correlacional Um questionário e três escalas	390 pessoas com ostomia (59,2% eram homens; e 60,2% deles tinham mais de 60 anos)	Avaliar a correlação entre a relação íntima com o cônjuge, a autorrevelação e a adaptabilidade em doentes com cancro colorretal e enterostomia.	O estudo destaca a aceitação como a dimensão mais valorizada pelos participantes. Por outro lado, a autorrevelação também se correlacionou positivamente com os níveis de adaptação, aceitação e uma atitude positiva perante a vida enquanto se correlaciona negativamente com a preocupação contínua. A maioria dos participantes (42,6%) referiu problemas nas relações íntimas conjugais, e 90,8% apresentavam níveis de adaptação baixos ou moderados. Preditores significativos para as relações íntimas conjugais incluíram formação escolar, capacidade de autocuidado e tempo pós-operatório. O estudo enfatiza a necessidade de educação em saúde para melhorar a autorrevelação e adaptabilidade entre doentes com cancro colorretal.
**Estudo 4**	McCarthy/2020/Canadá^(41)^	Paradigma qualitativo Estudo 1 – *Grounded Theory* (entrevista semiestruturada a casais) Apenas o estudo 1 cumpre os critérios de inclusão	11 casais (portador de ostomia idade entre 42 e 80 anos; cônjuge entre 37 e 76 anos)	Proporcionar um relato experiencial do ajustamento sexual e íntimo dos casais a uma colostomia permanente (após tratamento e cancro colorretal).	Os resultados evidenciam sofrimento emocional e preocupações identitárias significativas nas pessoas com cancro colorretal, reforçando a importância de cuidados de suporte proativos, por parte dos profissionais de saúde, para melhorar a sua qualidade de vida. Apesar do impacto dos tratamentos oncológicos e da ostomia no funcionamento sexual e na imagem corporal, os casais demonstram capacidade de permanecer unidos como uma díade e de resistir às alterações provocadas. Ainda que alguns relatem um fortalecimento da intimidade, persistem desafios físicos e psicossociais na adaptação sexual. Os resultados foram apresentados em duas categorias. Na categoria “Amar com um corpo imperfeito após uma colostomia”, destacam-se a importância da reconstrução da imagem corporal como facilitadora da aceitação do corpo e da integração da ostomia na sua identidade, o (des)conforto do parceiro e a influência da aceitação mútua na abertura à intimidade sexual. Já na categoria “Lidando com a função sexual depois do cancro”, destacam-se as implicações dos tratamentos na função e identidade sexual, o ritmo individual de cada membro do casal, a necessidade de pensar, globalmente, a comunicação conjunta, e a exploração de diferentes formas de intimidade.
**Estudo 5**	Tripaldi/2019/Irlanda^(42)^	Revisão da literatura	18 artigos - duas revisões de literatura e os restantes eram estudos de investigação (estudos qualitativos, quantitativos e mistos)	Explorar a experiência de sexualidade das mulheres após a formação de um estoma no decurso de uma cirurgia por cancro colorretal.	A revisão identificou três temas principais — perturbação da imagem corporal, relações íntimas e desvalorização da sexualidade —, evidenciando a insuficiência de educação pré-operatória na área da saúde sexual. As mulheres referiram sentir-se despreparadas para o impacto do estoma, enfrentando dificuldades na função sexual e na aceitação por parte do parceiro. A presença do estoma acentua as alterações na imagem corporal, contribuindo para uma redução da autoestima e da perceção de atratividade sexual associada à perda de libido, secura vaginal e limitações na participação em relações sexuais penetrativas. As mulheres tendem a antecipar a aceitação ou rejeição por parte do cônjuge, sendo ainda reportadas dificuldades conjugais. O apoio do parceiro revelou ser essencial para o ajustamento das mulheres, embora nem todos os parceiros tenham conseguido oferecer um suporte emocional adequado. Assim, torna-se evidente a importância de uma intervenção educativa e psicossocial integrada no período pós-operatório como estratégia fundamental para promover o bem-estar dessas mulheres.
**Estudo 6**	Alwi, Setiawanb & Asrizalb/2018/Indonésia^(43)^	Paradigma qualitativo Fenomenologia Entrevista	12 pessoas com ostomia	Descrever as experiências da pessoa com colostomia definitiva em relação à sua qualidade de vida.	O estudo identificou sete temas centrais na qualidade de vida de pessoas com colostomia permanente, nomeadamente: limitações nas atividades de vida diária; restrições nas relações conjugais e sociais; sentimentos negativos em relação à colostomia; dificuldades financeiras; exigências crescentes da vida; alterações das necessidades de repouso e complicações físicas; e esperança de viver uma vida normal após a colostomia. Em particular, as relações conjugais evidenciaram preocupações relativas ao divórcio e à cessação das relações sexuais, gerando receio e abstinência após a cirurgia. Na interação social, emergiram como principais obstáculos ansiedade, privacidade, diversas dificuldades práticas e traços de personalidade introvertida.
**Estudo 7**	Purba, Ibrahim & Rahayu/2018/Indonésia^(44)^	Paradigma qualitativo Fenomenologia Entrevista	Três pessoas com ostomia (idade entre 40 e 50 anos)	Obter a experiência significativa de sobreviventes de cancro rectal.	O estudo evidencia que os sobreviventes de cancro rectal com estoma vivenciam experiências que impactam a relação conjugal, sobretudo nas áreas da intimidade, sexualidade, apoio mútuo, gestão financeira e perda. A autoestima e imagem corporal ficam comprometidas, gerando vergonha, isolamento e dificuldade de proximidade com o parceiro. No plano sexual, todos relataram alterações na atividade, marcadas por medo de dor, rejeição ou desconforto, o que exige maior apoio profissional sensível ao contexto cultural. A perda da integridade física e do futuro idealizado é enfrentada com tristeza, aceitação progressiva e necessidade de adaptação, frequentemente suportada pela fé. Apesar das dificuldades, destaca-se o desejo de ajudar outros em situação semelhante, o que contribui para o fortalecimento emocional.
**Estudo 8**	McCarthy, Fergus & Miller/2016/Canadá^(45)^	Paradigma qualitativo (análise temática teórica) Entrevista	Nove casais (idade entre 42 e 80 anos)	Validar o Sistema de Classificação de Adaptação do Casal ao Cancro usando uma amostra de casais que se adaptaram ao cancro rectal e à colostomia permanente, e melhorar a estrutura com base nos dados emergentes da amostra.	O estudo constatou que todos os casais experimentaram flutuações entre o “eu” e o “nós” durante o processo de adaptação ao cancro do reto e à colostomia, evidenciando diferentes formas de afirmação e diferenciação do “nós”. Enquanto alguns participantes identificaram mudanças claras na relação e concordaram com a conceptualização de que há uma variação contínua entre ligação e separação, outros manifestaram uma reação mais mista, ainda que reconhecessem necessidades de espaço físico e emocional ao longo do percurso da doença. Apesar de o respeito pelas necessidades individuais não ser algo novo na dinâmica relacional, vários casais sublinharam ajustes específicos ligados às atividades da colostomia e ao tratamento do cancro, como a reorganização de responsabilidades práticas. Adicionalmente, alguns casais relataram momentos de proximidade e empatia reforçadas, reconhecendo que essas fases “de união” podem ser intercaladas com períodos de distanciamento ou isolamento, sem que isso prejudique de forma definitiva o vínculo conjugal. Pelo contrário, o reconhecimento mútuo de independência e autonomia se revelou fundamental para fortalecer o funcionamento coletivo e individual. Em síntese, as dinâmicas de afirmação e erosão do “nós”, evidenciadas ao longo do ajuste à doença, mostram-se naturais, não constituindo necessariamente um indicador de fracasso relacional. Pelo contrário, passar por esses desafios em conjunto reforça a resiliência do casal, intensificando a partilha, a empatia e uma maior identificação com o relacionamento.
**Estudo 9**	Persson, Severinsson & Hellström/2004/Suécia^(46)^	Paradigma qualitativo (análise de conteúdo) Entrevista de grupo	Nove cônjuges de pessoas com ostomia (dos quais seis eram mulheres, com idade entre 49 e 74 anos)	Conhecer as perceções dos cônjuges sobre o modo como vivem com um parceiro a quem foi diagnosticado cancro do reto seguido de uma cirurgia que resultou em uma ostomia.	Os principais resultados do estudo revelam que a experiência dos cônjuges de pessoas submetidas à ostomia por cancro colorretal é marcada por um conjunto de desafios. Os cônjuges, embora tentem apoiar os seus parceiros, relatam dificuldade em se envolver nos cuidados e no processo de informação, sentindo-se frequentemente excluídos e emocionalmente sós, uma vez que o foco da atenção recaía exclusivamente no doente. A incerteza sobre a evolução da doença e os receios quanto à recorrência do cancro geravam ansiedade constante, agravada pela falta de informação e pela perceção de não serem incluídos nas decisões clínicas. Após a cirurgia, foi necessário aprender a viver de uma nova forma, reorganizando a rotina familiar, os hábitos sociais e as estratégias de cuidado em função das exigências do estoma, implicando, assim, ajustamento familiar e adaptação à nova condição. A presença do estoma teve um impacto significativo na imagem corporal e na intimidade do casal, afetando a sexualidade e gerando sentimentos ambivalentes, como repulsa e culpa. Finalmente, os cônjuges procuravam explicações para a origem da doença, tentando encontrar sentido no sofrimento através da análise de fatores hereditários ou do estilo de vida do parceiro.

## RESULTADOS

Foram identificadas, através de pesquisa eletrónica em bases de dados e repositórios, 733 referências. Dessas, 105 foram encontradas na base de dados MEDLINE, 73, na CINAHL, quatro, na SciELO, nove, na *JBI Evidence Synthesis*, duas, na *Psychology and Behavioral Sciences Collection*, 19, na PsycINFO, 105, na *Web of Science*, 356, na Scopus, 37, na Cochrane, oito, na *Open Access Theses and Dissertations*, 13, na *ProQuest Dissertation and Theses Database*, e duas, no RCAAP. A pesquisa realizada no *Google Scholar* não devolveu resultados relevantes.

Dos 733 registos identificados, 710 foram provenientes das bases de dados, e 23 resultaram de outros métodos de pesquisa. Relativamente aos registos identificados nas bases de dados, 212 foram excluídos por estarem duplicados. Dos 498 registos restantes, 463 foram eliminados após leitura do título e do resumo, tendo sido selecionados 35 para recuperação em texto integral. Desses, um registo foi excluído devido à impossibilidade de acesso ao texto completo, apesar dos esforços desenvolvidos nesse sentido. A leitura integral dos 34 registos restantes resultou na exclusão de 26, por não cumprirem os critérios de inclusão, nomeadamente por a ostomia não decorrer de cirurgia por CCR, por a população incluir simultaneamente pessoas com e sem ostomia, por a população não corresponder ao casal, ou por abordarem um conceito distinto. No que diz respeito aos registos identificados por outros métodos, após a leitura do título e do resumo, três foram avaliados segundo os critérios de inclusão, tendo dois sido excluídos pela ostomia não decorrer de cirurgia por CCR e pela população incluir pessoas com e sem ostomia. Assim, um estudo proveniente de outros métodos foi incluído na revisão. No total, foram incluídos nesta revisão nove estudos (Figura 1).

Os estudos incluídos foram publicados entre 2004 e 2024, com pelo menos uma publicação por ano entre 2019 e 2024(38–42), à exceção de 2022, em que não se registaram publicações. Verificaram-se ainda duas publicações em 2018^(43,44)^ e uma publicação nos anos de 2016^(45)^ e 2004^(46)^. Relativamente à origem geográfica dos estudos, verificou-se a existência de três estudos realizados na Indonésia^(39,43,44)^, dois, no Canadá^(41,45)^, dois, na China^(38,40)^, um, na Irlanda^(42)^, e um, na Suécia^(46)^. Relativamente ao tipo de estudos incluídos, sete se enquadram no paradigma qualitativo, com quatro deles recorrendo à abordagem fenomenológica^(38,39,43,44)^, e um deles, à *Grounded Theory* ^(41)^, sendo que um utiliza a análise temática de orientação teórica^(45)^ e o outro utiliza a análise de conteúdo^(46)^. Um dos estudos se insere no paradigma quantitativo, de natureza correlacional^(40)^, e um corresponde a um estudo secundário, sob a forma de revisão da literatura^(42)^. No que diz respeito à população, cinco estudos integraram exclusivamente pessoas com ostomia^(38,39,40,43,44)^, e dois incluíram casais^(41,45)^, nos quais um dos elementos era portador de ostomia e um incluiu cônjuges de pessoas com ostomia^(46)^. Embora os critérios de elegibilidade definidos tenham considerado como adultos as pessoas com idade igual ou superior a 19 anos, alguns estudos consideram como adultos pessoas com 18 ou mais anos. Assim, a idade dos participantes envolvidos variou entre 18 e 80 anos.

Relativamente aos conceitos que são utilizados na evidência acedida, para descrever a experiência do casal perante uma OEI por CCR, foram identificados os conceitos de adaptação e ajustamento.

Foi realizada análise de conteúdo qualitativa de natureza descritiva dos resultados dos estudos incluídos. Procedeu-se a uma leitura minuciosa e iterativa dos dados, a partir da qual foram identificados, de forma indutiva, temas que caracterizassem a experiência do casal. Esses temas foram posteriormente agrupados, por similitude, em categorias e subcategorias. O processo de análise foi conduzido manualmente, sem recurso a *software* específico de apoio à análise qualitativa, tendo sido discutido entre os investigadores até se alcançar consenso quanto à definição e organização final das categorias.

A análise dos dados extraídos permitiu caracterizar a experiência do casal que enfrenta uma OEI por CCR em três categorias: natureza da experiência; fatores influenciadores; e resultados decorrentes dessa experiência (Quadro 3).

**Quadro 3:** Caraterização da experiência do casal – Lisboa, Portugal, 2025.

Dimensões		Dados
**Natureza da experiência**		Desafio constante à medida que lidam com novas e antigas preocupações físicas e psicossociais^(41,46)^ Sentimento de desconexão “no mesmo espaço, mas não conectado” à sua parceira^(45)^ Flutuação entre o “eu” e o “nós”^(45)^ Inevitabilidade de períodos de isolamento do cônjuge^(45)^ Vários processos de afirmação e diferenciação do “nós”^(45)^
**Fatores**	**Facilitadores**	Apoio interdisciplinar^(38–40,42)^ e aconselhamento pré e pós-cirúrgico^(39,40,42)^ Cuidados colaborativos^(38,39,45,46)^ e redistribuição de responsabilidades^(45,46)^ Apoio/suporte do cônjuge^(39,41,42,46)^ Cuidados de suporte pelos profissionais de saúde^(41)^ Capacidade de autocuidado^(40,42)^ Comunicação em casal^(39,41)^ e em sociedade (comunidade)^(41)^ Pensar em termos globais^(41)^ Definição de objetivos conjuntos, reconhecimento sincero e encorajamento^(38,46)^ Informação, acolhimento dos profissionais de saúde e a sua capacidade de resposta^(44)^ Atitude positiva perante a vida^(40,46)^ Esperança de viver uma vida normal após a ostomia^(43,46)^ Confiança mútua^(38)^ Compreensão mútua, empatia e vivência interpessoal^(45)^ Recorrer ao senso de “nós” para superar a adversidade^(45)^ Toque não sexual^(38)^ Contacto com a natureza^(38)^ Condição socioeconómica estável^(41)^ Fé^(38)^
	**Inibidores**	Sentimentos negativos em relação à ostomia, como medo^(39,43,44,46)^, insegurança^(43,44,46)^, ansiedade^(38,43,46)^, depressão^(38)^, vergonha^(38,43)^, preocupação^(38,40–44)^, estresse psicológico^(38,42)^ Falta de informação e educação para a saúde^(38,40–42,46)^ Diminuição da libido^(38,39,42,43,46)^ Diminuição da atratividade^(38,42,44,46)^ Falta de comunicação e apoio dos profissionais^(39,42)^ Falta de demonstração de interesse pelo cônjuge^(38)^ Dificuldades financeiras^(43,44)^
**Resultados**	**Positivos**	Aceitação da nova condição^(38,40–42,45,46)^ Melhoria da comunicação em casal^(38,39,41–43)^ e com a sociedade/comunidade^(41)^ Apoio e interação conjugal e social^(38,41,42,45,46)^ Cuidados colaborativos entre o casal^(38,39,45,46)^ Capacidade de resistência às mudanças sexuais^(41)^ Desenvolvimento enquanto casal^(39,41,45)^ Sentimento de união^(41,46)^ Tornar-se mais próximos^(39,45)^ Enfrentar e reparar a autoimagem^(38,41,46)^ Enfrentar o impacto da ostomia e do tratamento oncológico no relacionamento sexual^(45)^ Reformulação da identidade após a ostomia^(41)^ Preservação da identidade do “eu” pelo outro^(41)^ Celebração e expansão do reportório sexual^(41)^ Reforço das ligações emocionais e espirituais em detrimento da intimidade sexual^(38)^ Cura espiritual^(38)^ Redescobrir valor pessoal e o significado da vida^(38)^
	**Negativos**	Limitações nas atividades diárias, nas relações conjugais^(38, 41,43,44)^ e na vida sexual^(38,39,42–44)^ Mudanças nas necessidades físicas e de repouso^(42–44)^ Alterações no estilo e padrão sexual^(39,42,44,46)^ Desgaste no relacionamento^(38,42,46)^ Isolamento social^(38,43,44,46)^ Baixa satisfação^(38,39,44)^ e baixa autoestima^(42,44,46)^ Alteração da identidade^(38)^ Sofrimento emocional significativo^(41)^

No que diz respeito à natureza da experiência, os estudos analisados descrevem um conjunto de desafios e transformações vividos pelos casais. Foi reportado que esta experiência se configura um desafio constante, marcado pela convivência simultânea com preocupações físicas e psicossociais tanto recentes quanto anteriores^(41,46)^. Foram identificados sentimentos de desconexão entre os cônjuges, expressos pela perceção de “estar no mesmo espaço, mas não conectado”^(45)^, bem como uma flutuação entre a individualidade (“eu”) e a identidade relacional (“nós”)^(45)^. Relatam-se, ainda, processos de diferenciação e afirmação do “nós” conjugal^(45)^, bem como a ocorrência inevitável de períodos de isolamento entre os membros do casal^(45)^.

Entre os fatores facilitadores do processo de adaptação, destacaram-se o apoio interdisciplinar^(38,39,40,42)^ e o aconselhamento pré e pós-cirúrgico^(39,40,42)^. A prestação de cuidados colaborativos^(38,39,45,46)^ e a redistribuição de responsabilidades no seio do casal^(45,46)^ foram igualmente relevantes. O suporte do cônjuge^(39,41,42,46)^ e os cuidados de suporte prestados pelos profissionais de saúde^(41)^ surgiram como determinantes, a partir da capacidade de autocuidado^(40,42)^, da comunicação conjugal^(39,41)^ e da interação em comunidade^(41)^. Outros elementos considerados facilitadores incluíram o pensamento em termos globais^(41)^, a definição de objetivos conjuntos e o encorajamento mútuo^(38,46)^, a disponibilidade informativa e o acolhimento por parte dos profissionais de saúde^(44)^, bem como uma atitude positiva perante a vida^(40,46)^, a esperança de retomar uma vida normal após a ostomia^(43,46)^, a confiança mútua^(38)^, a empatia e compreensão interpessoal^(45)^, o recurso ao senso de “nós” para ultrapassar a adversidade^(45)^, o toque não sexual^(38)^, o contacto com a natureza^(38)^, a condição socioeconómica estável^(41)^, e a fé^(38)^.

Por outro lado, como fatores inibidores, foram salientados sentimentos negativos associados à ostomia, tais como medo^(39,43,44,46)^, insegurança^(43,44,46)^, ansiedade^(38,43,46)^, depressão^(38)^, vergonha^(38,43)^, preocupação^(38,40,41,42,43,44)^ e estresse psicológico^(38,42)^. A falta de informação e de educação para a saúde^(38,40,41,42,46)^, a diminuição da líbido^(38,39,42,43,46)^, a perceção de diminuição da atratividade^(38,42,44,46)^, a ausência de comunicação e apoio por parte dos profissionais de saúde^(39,42)^, a falta de demonstração de interesse pelo cônjuge^(38)^, e as dificuldades financeiras^(43,44)^ foram também identificadas como barreiras relevantes ao processo de adaptação.

A análise dos estudos permite, ainda, identificar um conjunto de resultados tanto positivos quanto negativos. Relativamente aos resultados positivos, foram reportados a aceitação da nova condição de vida^(38,40,41,42,45,46)^, a melhoria da comunicação entre os elementos do casal^(38,39,41,42,43)^ e com a comunidade^(41)^, o reforço do apoio conjugal e social^(38,41,42,45,46)^, e a implementação de cuidados colaborativos no seio da relação^(38,39,45,46)^. Verificaram-se também a capacidade de resistência às alterações na vida sexual^(41)^, o desenvolvimento enquanto casal^(39,41,45)^, o fortalecimento do sentimento de união^(41,46)^ e a perceção de maior proximidade entre os parceiros^(39,45)^. Foram igualmente identificados processos de enfrentamento e reparação da autoimagem^(38,41,46)^, de superação dos impactos da ostomia e do tratamento oncológico na sexualidade^(45)^, de reformulação da identidade após a ostomia^(41)^, e de preservação da identidade do “eu” pelo outro^(41)^. Adicionalmente, foram referidas experiências de celebração e expansão do reportório sexual^(41)^, reforço das ligações emocionais e espirituais em detrimento da intimidade sexual^(38)^, cura espiritual^(38)^, e redescoberta do valor pessoal e do significado da vida^(38)^.

Por fim, quanto aos resultados negativos, foram referidas limitações nas atividades da vida diária, nas relações conjugais^(38,41,43,44)^ e na vida sexual^(38,39,42,42,44)^, alterações nas necessidades físicas e de repouso^(42,43,44)^, bem como modificações no estilo e padrão sexual^(39,42,44,46)^. Foram ainda identificados indícios de desgaste relacional^(38,42,46)^, isolamento social^(38,43,44,46)^, baixa satisfação conjugal^(38,39,44)^, baixa autoestima^(42,44,46)^, alterações da identidade^(38)^ e sofrimento emocional significativo^(41)^.

Para além disso, as lacunas identificadas na evidência se referem à escassez de estudos primários que: i) se dediquem à experiência do casal perante uma OEI por CCR; ii) incluam como participantes a díade conjugal; iii) descrevam a dinâmica da experiência destes casais, nomeadamente os processos de mudança vivenciados; e iv) abordem a experiência do casal como um processo de transição múltipla — de saúde/doença e situacional — vivido pelo casal.

## DISCUSSÃO

Os resultados traduzem a existência de um reduzido número de estudos sobre a experiência do casal que enfrenta uma OEI por CCR, com destaque para estudos que se focam na sexualidade do casal (cinco dos nove estudos)^(38,42,42)^, privilegiando, na maioria dos casos, a perspectiva individual. Essa escassez de estudos denota que esse fenómeno ainda está pouco estudado, apontando para necessidade de serem desenvolvidos estudos primários. Na evidência acedida, apenas dois estudos tiveram como participantes diretos o casal^(41,45)^, sendo que os restantes acederam aos dados sobre a experiência do casal através da pessoa ostomizada^(38,39,40,43,44)^ ou do cônjuge^(46)^. Esta opção metodológica pode comprometer a compreensão da vivência conjugal da OEI por CCR, uma vez que fenómenos relacionais são, por natureza, interdependentes. Como referem Revenson *et al.* ^(47)^, fenómenos com elevado impacto funcional e simbólico, como uma ostomia decorrente de CCR, devem ser compreendidos enquanto experiências partilhadas, o que exige abordagens metodológicas capazes de captar a reciprocidade dos significados, das emoções e das estratégias de enfrentamento no seio da díade conjugal. Nesse sentido, diversos autores^(48,50,50)^ sublinham que o recurso a dados diádicos permite aceder de forma mais fidedigna à complexidade da relação conjugal. Quando apenas um dos elementos do casal é incluído no estudo, os dados obtidos podem refletir percepções parciais, enviesadas por fatores emocionais, limitações narrativas ou dificuldades em expressar vivências relacionais de forma abrangente.

A análise dos objetivos dos estudos incluídos nesta *scoping review* permitiu verificar que, de forma geral, estes se centram na exploração da experiência do casal perante o impacto da ostomia associada ao CCR, com particular ênfase na sexualidade do casal, na qualidade de vida e nos desafios relacionados à adaptação e ao ajustamento à nova condição. Os estudos incluídos reconhecem diversas mudanças experienciadas pelos casais que vivem com uma OEI decorrente de CCR, designadamente nas dimensões física, emocional, relacional, social e espiritual.

Os estudos que integraram esta *scoping review* utilizam os conceitos de adaptação e de ajustamento quando se referem à experiência. Ainda que utilizados com frequência, esses conceitos raramente são acompanhados de definições, o que dificulta a sua compreensão, distinção analítica e comparação entre estudos. Não obstante, com base na análise realizada, depreende-se que a adaptação é multidimensional^(38,41,42,45)^, envolvendo a resposta e a integração às novas circunstâncias. É indispensável para a manutenção do bem-estar emocional, físico e psicológico do casal, e da qualidade da relação conjugal^(38,41,42,45)^. Já ajustamento corresponde a uma trajetória contínua de resposta prática às mudanças físicas, psicológicas e sociais impostas pela condição^(39,43,45)^. De forma geral, o ajustamento é descrito como as estratégias adotadas para lidar com as disrupções sexuais após a cirurgia^(39)^, as modificações realizadas nas rotinas diárias, nos cuidados pessoais e nas interações sociais^(43)^, e em uma perspectiva relacional, implica equilíbrio delicado entre a preservação da identidade individual de cada elemento e a manutenção da ligação afetiva e funcional da díade^(45)^. A evidência acedida permite perceber que esses conceitos estão interligados. No estudo de Tripaldi^(42)^, os conceitos de adaptação e ajustamento estão presentes de forma implícita, mas não são apresentados de forma independente nem diferenciados conceptualmente, surgindo integrados na discussão de estratégias de *coping*, alterações na identidade e dinâmicas relacionais. Contudo, uma análise mais detalhada revela que a adaptação é concebida como uma resposta — englobando integração e aceitação^(40,43)^ —, enquanto o ajustamento reflete um processo ativo e dinâmico de mudança e negociação^(45)^.

Importa ainda destacar que nenhum dos estudos recorreu explicitamente ao conceito de transição para designar a experiência vivida pelo casal que enfrenta uma OEI no contexto do CCR. Sendo a transição definida como um processo e resultado da passagem de uma condição ou estado para outro, designadamente os relacionados à saúde e ao desempenho de papéis^(24,25)^, seria expectável que tal conceito fosse utilizado na investigação da experiência desses casais. Essa ausência evidencia uma fragilidade relevante na abordagem teórica da problemática em estudo, e sugere a pertinência de aprofundar a compreensão dessa vivência à luz da perspectiva da Teoria das Transições, contribuindo, assim, para uma análise mais abrangente e sensível às dinâmicas processuais envolvidas.

Em relação à natureza da experiência, os estudos salientam os desafios e as mudanças experienciados pelos casais. Vários autores^(41,46)^ referem que essa vivência constitui um desafio constante, uma vez que os casais lidam simultaneamente com preocupações emergentes relacionadas à doença e à ostomia, e com questões psicossociais previamente existentes, que podem ser reativadas ou aumentadas no contexto da adversidade. Contudo, revelam que a experiência é vivida de forma divergente entre os casais estudados, oscilando entre sentimentos de desconexão conjugal^(45)^ e reforço do vínculo^(39,45)^ entre o “eu” e o “nós”, revelando a complexidade da adaptação a uma nova identidade, condicionada pela ostomia e pelas repercussões da doença.

A evidência científica demonstra que essa alternância entre desconexão e fortalecimento conjugal^(21)^, no contexto de uma ostomia, não ocorre de forma aleatória, mas resulta da interação complexa entre múltiplos fatores relacionais. Nesse sentido, a comunicação eficaz assume um papel decisivo: os casais que conseguem partilhar emoções, negociar papéis e trocar informações com abertura tendem a reforçar o sentimento de pertença e de unidade (“nós”)^(51)^. Esses autores identificam que a eficácia diádica, ou seja, a confiança conjunta na capacidade de enfrentar o cancro, é facilitada por uma comunicação fluida, interesse mútuo na informação e estratégias de *coping* comuns, enquanto as barreiras nessa comunicação resultam em menor coesão e maior sofrimento emocional.

O alinhamento de expectativas e perceções entre os cônjuges também se mostra fundamental. Qin *et al.* ^(52)^ salientam que abordagens congruentes na perceção do estresse e nas respostas adaptativas, nomeadamente entre medo da recidiva e abordagem conjunta, contribuem para níveis superiores de resiliência e ajustamento conjugal.Quando as perceções divergem, por exemplo, quando a pessoa ostomizada minimiza o impacto enquanto o cônjuge se sente sobrecarregado, tende a surgir uma crescente sensação de desconexão e desajuste na díade.

Adicionalmente, o apoio social e o acesso a recursos profissionais, como programas de *coping* para casais com CCR, demonstram impactar positivamente a experiência partilhada. Intervenções estruturadas que promovem a comunicação de estresse e o *coping* comunitário, bem como estratégias educativas destinadas a envolver ambos os parceiros, têm sido associadas a melhorias na satisfação relacional e na saúde mental individual^(51,53,54)^. Nesta linha, e de acordo com os dados obtidos nesta revisão, depreende-se que fatores como boa comunicação, partilha de responsabilidades, alinhamento emocional e suporte profissional adequado favorecem a emergência de um renovado sentido de “nós”. Esse sentido partilhado permite aos casais enfrentar a ostomia e as repercussões do CCR com maior coesão e ajustamento mútuo. Por oposição, os fatores inibidores apontam para barreiras à adaptação, nomeadamente sentimentos de vergonha, medo, ansiedade e depressão, falta de informação, apoio profissional, e dificuldades económicas.

Os resultados da revisão, ao identificarem como fator facilitador da experiência o apoio por parte dos profissionais de saúde, e, como fatores inibidores, a escassez de informação e de educação para a saúde, bem como a ausência de uma comunicação profissional de apoio, parecem evidenciar a necessidade dos profissionais — nomeadamente os enfermeiros — aprofundarem o seu conhecimento sobre esse fenómeno, de modo a fundamentar intervenções que apoiem efetivamente os casais perante essa experiência.

A leitura crítica dos resultados permite constatar que a vivência do casal perante uma OEI por CCR é simultaneamente transformadora e vulnerável, integrando dimensões positivas, como aceitação, apoio mútuo, colaboração nos cuidados e reforço da identidade conjugal, e dimensões negativas, como alterações na intimidade sexual, isolamento emocional, e impacto na autoestima e na identidade individual. Essa coexistência de polaridades sugere que a experiência da ostomia por CCR, quando vivida no contexto conjugal, não é linear e nem unívoca, e confirma que enfrentar uma OEI por CCR ultrapassa os limites da adaptação individual, constituindo uma experiência relacional que envolve a renegociação de papéis e de significados no seio da díade conjugal. Essa perspectiva encontra suporte na literatura recente. Lin *et al.* ^(55)^, por exemplo, sublinham que as alterações funcionais e as perturbações psicológicas interferem significativamente na intimidade conjugal e na percepção do valor pessoal, reforçando a importância de intervenções orientadas para a relação diádica. Nesse sentido, os dados desta revisão evidenciam que os resultados positivos e negativos da experiência conjugal coexistem e interagem, sustentando a necessidade de estratégias de intervenção específicas centradas no casal, como aconselhamento sexual e conjugal, programas educativos para casais e apoio emocional continuado, que favoreçam os processos de ajustamento mútuo^(53,54)^.

### Limitações

Identificam-se como limitações desta *scoping review* ter integrado um número reduzido de estudos que abordam a experiência do casal que enfrenta uma OEI por CCR como unidade. Embora a evidência privilegie a experiência do casal, o facto de a maioria dos estudos selecionados ter como participantes apenas um membro ostomizado daquele, poderá ter gerado algum viés de perceção nos resultados daqueles estudos e, por tal, também nos desta revisão. Acresce uma limitação de ordem metodológica, relacionada à impossibilidade de aceder ao texto completo de um dos artigos identificados durante o processo de seleção. Apesar dos esforços, nomeadamente a consulta a repositórios institucionais, bibliotecas académicas e o contacto direto com os autores, não foi possível garantir o seu acesso, o que restringiu o corpo de evidência disponível para análise.

### Contribuições para a Área da Enfermagem

Apesar de a natureza desta revisão não permitir retirar implicações para a prática, os resultados apontam para elementos importantes que podem orientar os profissionais de saúde, concretamente os enfermeiros. Os resultados desta *scoping review* alertam para exigências e transformações que os casais podem enfrentar nesta condição, evidenciando a importância de assegurar suporte contínuo e educação para a saúde, mediante intervenções que facilitem os processos de mudança enfrentados pelo casal, promovendo, entre outros resultados, a resiliência e o bem-estar conjugal.

## CONCLUSÃO

A presente revisão, sem limite temporal, permite concluir a escassez de evidência científica sobre o fenómeno. A evidência disponível teve origem em seis países. Embora reportando à experiência do casal perante uma EOI por CCR, os participantes foram maioritariamente a pessoa com ostomia. Na nomeação daquela experiência, destacou-se o recurso aos conceitos de adaptação e de ajustamento. A experiência desses casais foi marcada pela necessidade de ajustes contínuos e mútuos perante as mudanças impostas pela ostomia e por uma dualidade entre desafios e oportunidades de crescimento. Tal experiência foi caracterizada em três categorias: i) a natureza da experiência; ii) fatores facilitadores e inibidores; e iii) resultados positivos e negativos.

Centrando a atenção na construção de conhecimento relevante para a enfermagem, considera-se importante o desenvolvimento de investigação primária que possibilite melhorar a compreensão da experiência dos casais que enfrentam OEI por CCR. Nesse âmbito, destaca-se a relevância do estudo da dinâmica subjacente à sua adaptação, ajustamento ou transição diante de tais condições, ou seja, o estudo do(s) processo(s) de mudança enfrentado(s) pela díade. Os achados destes estudos poderão constituir uma base de conhecimento para o desenvolvimento posterior de intervenções que facilitem tais processos e a obtenção de resultados em saúde para esses casais.

## Data Availability

Todo o conjunto de dados que dá suporte aos resultados deste estudo está disponível mediante solicitação ao autor correspondente.

# Translation: Experiencia de la pareja en caso de ostomía por cáncer colorrectal de uno de los miembros: *revisión de alcance*

**DOI:** 10.1590/1980-220X-REEUSP-2025-0250es

**Authors:** Ricardo Emanuel Sousa Mestre, Ana Beatriz Gomes Dias, Célia Maria Gonçalves Simão de Oliveira, Patrícia Vinheiras Alves

**Affiliations:** 1 Escola Superior de Saúde Atlântica, Lisboa, Portugal.; 2 Instituto Português de Oncologia de Lisboa Francisco Gentil, Lisboa, Portugal.; 3 Universidade de Lisboa, Escola Superior de Enfermagem, Lisboa, Portugal.; 4 Universidade de Lisboa, Escola Superior de Enfermagem, Centro de Investigação Inovação e Desenvolvimento em Enfermagem de Lisboa, Lisboa, Portugal.

**Keywords:** Adaptación Psicológica, Ajuste Social, Neoplasias Colorrectales, Composición Familiar, Estomía

## Abstract

**Objetivo:**

Mapear la evidencia científica sobre la experiencia compartida de parejas sometidas a ostomía intestinal por cáncer colorrectal.

**Método:**

Se realizó una revisión del alcance siguiendo las directrices de la JBI. La búsqueda se llevó a cabo en 13 bases de datos y repositorios, con criterios de inclusión definidos por el enfoque PCC (Participantes, Concepto y Contexto).

**Resultados:**

Se incluyeron nueve estudios, lo que nos permitió mapear la experiencia de la pareja y resumir sus características según la naturaleza, los factores influyentes y los resultados de esta experiencia; al nombrar esta experiencia, predominaron los conceptos de adaptación y/o ajuste.

**Conclusión:**

La experiencia de las parejas con ostomía por cáncer colorrectal varía entre ellas e implica desafíos físicos, emocionales y relacionales. Se identifican lagunas en la evidencia científica, lo que subraya la necesidad de investigación primaria que permita comprender mejor esta experiencia, en particular la dinámica o el proceso que viven estas parejas.

## INTRODUCCIÓN

A nivel mundial, el cáncer colorrectal (CCR) fue el tercer tipo de cáncer más diagnosticado en 2020, ocupando el tercer lugar en términos de incidencia y el segundo en términos de mortalidad^(1)^. A pesar de su gravedad, en los últimos años se ha observado una reducción en la tasa de mortalidad por CCR atribuida a los avances en el diagnóstico precoz y el tratamiento^(2)^. La cirugía es el tratamiento de elección^(3,4,5)^, lo que a menudo da lugar a la construcción de una ostomía de eliminación intestinal (OEI) provisional o definitiva^(6)^. Aunque no existen datos globales precisos, se estima que alrededor de un millón de personas en Estados Unidos^(7,8)^, un millón en China y 700.000 en Europa viven actualmente con por lo menos una ostomía^(7)^, y es de esperar que esta cifra siga aumentando como consecuencia del crecimiento de la incidencia del CCR^(9)^.

El diagnóstico y el tratamiento del CCR representan una experiencia profundamente desafiante, marcada por el sufrimiento físico y emocional, la tristeza, la ansiedad, el miedo y la inseguridad^(10)^. La presencia de una OEI agrava los cambios que experimenta la persona, generando desafíos adicionales relacionados con la imagen corporal, la autoestima, la sexualidad y la participación en actividades sociales, físicas y de ocio^(11,12)^. Estos cambios afectan directamente a la vida cotidiana y a la percepción de la calidad de vida^(11,12,13)^.

Partiendo del principio de que los acontecimientos vividos por un miembro de la familia afectan al resto^(14)^, es plausible afirmar que, en el contexto de una relación conyugal, estos cambios son experimentados por la pareja como unidad. Se considera pareja a la unión de dos personas adultas que mantienen una relación significativa, íntima y duradera^(15)^, compartiendo un proyecto de vida común^(16)^. En este contexto, el cónyuge asume a menudo el papel de cuidador principal, enfrentándose también a cambios sustanciales en su vida cotidiana, con repercusiones emocionales, sociales y relacionales^(17,18)^. Así, se entiende que la experiencia vivida por la pareja en respuesta al CCR y a la OEI se refleja en reajustes conjuntos, con un impacto significativo en la relación conyugal, así como en la salud mental y física de ambos cónyuges^(18,19,20)^.

La experiencia de la pareja que se enfrenta a un reto de esta naturaleza se reconoce como un proceso dinámico y evolutivo, caracterizado por transiciones continuas^(20)^, adaptaciones mutuas^(20,21,22)^y ajustes conyugales^(20,23)^. Esta perspectiva subraya que la experiencia de la pareja está en constante transformación, lo que se refleja en la forma en que los cónyuges se adaptan y se reorganizan ante los cambios impuestos por la condición de salud.

En este contexto, los cambios provocados por el CCR y la OEI desencadenan un proceso que puede conceptualizarse como transición, entendida como el paso de una condición o estado a otro^(24,25)^. La transición, como experiencia humana y dinámica, exige que la persona o, en el caso que nos ocupa, la pareja se involucre en procesos de adaptación y ajuste^(24,25,26)^. Este proceso es complejo y se produce a lo largo del ciclo vital, desencadenado por acontecimientos críticos —en particular los relacionados con la salud y el desempeño de roles— y que da lugar a cambios de roles, la adquisición de nuevos conocimientos, cambios en esos roles y la adaptación de comportamientos^(25,27)^.

Así, la adaptación y el ajuste surgen como elementos centrales de ese proceso, influyendo en la capacidad de la pareja para reorganizar su vida ante la nueva condición^(27,28)^. La adaptación se refiere a la capacidad de reorganizar su vida e identidad, integrando las nuevas exigencias impuestas por la condición de salud y promoviendo el bienestar, la continuidad de la relación conyugal y la relación con el mundo exterior^(29)^. El ajuste, por su parte, se refiere a la participación activa de la pareja, condicionada por el conocimiento que ambos tienen de su propia situación y por la capacidad de gestionar los cambios e integrar la nueva condición de vida^(27,30)^. De este modo, la adaptación se traduce en la integración de los cambios en la vida cotidiana, mientras que el ajuste refleja el proceso continuo de gestionar y afrontar esos cambios.

La pareja que se enfrenta a una OEI por CCR constituye un grupo particular en lo que respecta a las necesidades de atención médica^(17)^. Sin embargo, la evidencia científica se ha centrado predominantemente en la experiencia individual de la persona con OEI, del cónyuge como cuidador o de la familia en general, descuidando la comprensión de la experiencia compartida de la pareja como unidad. Esta laguna es especialmente relevante, ya que la experiencia de la OEI por CCR no se limita a la persona afectada, sino que abarca a la pareja, influyendo en su relación, comunicación, roles y bienestar emocional. La enfermería, como disciplina que privilegia la atención centrada en la persona, tiene la responsabilidad de comprender esta experiencia compartida, promoviendo intervenciones que respondan a las necesidades de la pareja como unidad^(27,31,32)^. Una investigación preliminar en las bases de datos JBI Evidence Synthesis, Cochrane, CINAHL, MEDLINE, PROSPERO, Open Science Framework y Figshare, utilizando los términos de búsqueda “transición”, “adaptación”, “ajuste”, “neoplasias colorrectales”, “ostomía” y “pareja”, en los idiomas portugués, inglés, francés y español, no identificó ninguna revisión de la literatura, publicada o en curso, que se centrara en el análisis de la pareja como unidad participante en el proceso de vivir una OEI por CCR.

En este sentido, se justifica la realización de esta revisión de alcance, cuyo objetivo es mapear^(33,34,35)^la evidencia científica sobre la experiencia conjunta de la pareja que se enfrenta a una OEI por CCR, identificando la evidencia científica disponible y las lagunas en este conocimiento, con el fin de fundamentar futuras investigaciones que contribuyan al desarrollo del conocimiento en enfermería en relación con este fenómeno en esta población.

## MÉTODO

### Tipo De Estudio

Se trata de una revisión de alcance realizada según la metodología propuesta por el JBI^(33,34)^y las directrices de la lista de verificación Preferred Reporting Items for Systematic Reviews and Meta-Analyses extension for Scoping Reviews (PRISMA-ScR)^(34,36)^. El protocolo se registró previamente en la plataforma Open Science Framework, con el DOI: 10.17605/OSF.IO/E8WJD. Durante el desarrollo de la revisión, se constató que era necesario introducir cambios en el protocolo: se reformuló la terminología utilizada en el título, el objetivo y las preguntas de la revisión, debido a que se observó que los artículos seleccionados no utilizaban el concepto de transición para nombrar la experiencia de las parejas.

Se definió como pregunta de revisión “¿Cuál es la evidencia científica disponible sobre la experiencia conjunta de la pareja que se enfrenta a una OEI por CCR?”, así como las subpreguntas “¿Cuáles son los estudios existentes sobre la experiencia de la pareja ante una OEI por CCR?”, “¿Cuáles son las características de la población en la evidencia disponible sobre esta experiencia?”, “¿Qué conceptos se utilizan para describir la experiencia de la pareja?” y “¿Cómo se caracteriza, en la evidencia disponible, la experiencia de la pareja?”.

### Criterios de Selección

Los criterios de elegibilidad se definieron basándose en el mnemotécnico PCC (Participantes, Concepto y Contexto), tal y como lo describen Peters *et al.* ^(33,34)^. En lo que respecta a los participantes, la revisión consideró estudios que incluían a adultos (edad ≥ 19 años) en una relación conyugal, en la que uno de los miembros era portador de OEI debido a CCR, en los que los datos se recopilaron junto con la pareja, la persona con ostomía o el cónyuge. A efectos de elegibilidad, se consideró como “pareja” cualquier referencia explícita en los estudios a términos como “pareja”, “compañero”, “cónyuge” o “esposo”, independientemente del género o estado civil. Se excluyeron los estudios en los que la OEI no se había construido como consecuencia de un CCR.

En cuanto al concepto, se incluyeron estudios que abordaban la experiencia de la pareja ante la OEI resultante de un CCR, así como estudios que investigaban la transición, la adaptación o el ajuste de la pareja ante esta condición. Se excluyeron los estudios que no se centraban en la experiencia, la transición, el ajuste o la adaptación de la pareja, así como aquellos que exploraban estos conceptos solo a nivel individual, centrándose exclusivamente en la persona con ostomía o en el cónyuge. En cuanto al contexto, no se impusieron restricciones relacionadas con factores culturales, étnicos, de género o de ubicación geográfica.

La revisión incluyó estudios primarios, secundarios y una tesis. En los estudios seleccionados, se identificó una revisión de la literatura. Sin embargo, no se verificó ninguna duplicación de datos derivada de la inclusión de estudios primarios en esta revisión, por lo que no fue necesario excluir dichos estudios primarios^(34)^. Debido al propósito de la revisión, se excluyeron libros, capítulos de libros y artículos de opinión. Se consideraron estudios publicados en inglés, portugués, francés y español, sin aplicar ninguna restricción temporal.

### Recopilación de Datos

La búsqueda inicial se realizó entre mayo y junio de 2024 y se actualizó en julio de 2025, siguiendo un enfoque multifásico, con el objetivo de identificar el mayor número posible de estudios y publicaciones relevantes. En la primera fase, se realizó una búsqueda en las bases de datos electrónicas MEDLINE (a través de EBSCO), CINAHL (a través de EBSCO), SciELO, Cochrane Database of Systematic Reviews (a través de EBSCO), JBI Evidence Synthesis, Psychology and Behavioral Sciences Collection (a través de EBSCO), PsycINFO (a través de EBSCO), Web of Science y Scopus. En la segunda fase, con el objetivo de integrar la literatura gris, se realizaron búsquedas en Open Access Theses and Dissertations, ProQuest Dissertation and Theses Database, Repositório Científico de Acesso Aberto de Portugal (RCAAP) y Google Scholar. No fue posible incluir datos procedentes de OpenGrey y DART-Europe, ya que estaban inactivos en la fecha de recopilación de la información. Por último, en la tercera fase, se procedió al análisis de las referencias bibliográficas de los estudios incluidos, con el fin de identificar artículos adicionales potencialmente relevantes para el estudio.

Los términos de los *Medical Subject Headings* identificados previamente se incluyeron y adaptaron a cada fuente de información utilizada, desarrollando una estrategia de búsqueda completa con operadores de truncamiento y booleanos apropiados (Cuadro 1).

**Cuadro 1:** Estrategia de búsqueda – Lisboa, Portugal, 2025.

Base de datos	Estrategia de búsqueda
CINAHL *complete* (EBSCO)	(XB (caregiv* OR “care partner” OR conjugal OR coupl* OR dyad* OR “family member” OR husband* OR “life partner” OR marital* OR married* OR mutuality OR partner* OR spous* OR wife OR wive) OR MH (caregivers OR marriage OR “married women” OR “married men” OR spouses OR “significant other”)) AND (XB (stoma* OR enterostomy OR ostomy OR ileostomy OR colostomy) OR MH ostomy+) AND (XB (“colorectal cancer” OR “CRC” OR “bowel cancer” OR “colon cancer” OR “rectal cancer”) OR MH (“cancer patients” OR “intestinal neoplasm+” OR neoplasms)) AND (XB (Change* OR experience* OR “life-change event” OR “life changing event” OR “life-changing event” OR “life change*” OR transition* OR adjustment* OR adaptation) OR MH (“life change events+” OR “adaptation, psychological+” OR “life experiences+” OR “social adjustment”))
MEDLINE *Complete* (EBSCO)	(XB (caregiv* OR “care partner” OR conjugal OR coupl* OR dyad* OR “family member” OR husband* OR “life partner” OR marital* OR married* OR mutuality OR partner* OR spous* OR wife OR wive) OR MH (caregivers OR marriage OR spouses)) AND (XB (stoma* OR enterostomy OR ostomy OR ileostomy OR colostomy) OR MH (ostomy+ OR “surgical stomas”)) AND (XB (“colorectal cancer” OR “CRC” OR “bowel cancer” OR “colon cancer” OR “rectal cancer”) OR MH (“intestinal neoplasms+” OR neoplasms)) AND (XB (Change* OR experience* OR “life-change event” OR “life changing event” OR “life-changing event” OR “life change*” OR transition* OR adjustment* OR adaptation) OR MH (“life change events” OR “adaptation, psychological+” OR “social adjustment”))
*Psychology & Behavioral Sciences Collection* (EBSCO)	(XB (caregiv* OR “care partner” OR conjugal OR coupl* OR dyad* OR “family member” OR husband* OR “life partner” OR marital* OR married* OR mutuality OR partner* OR spous* OR wife OR wive) OR SU (caregiv* OR “care partner” OR conjugal OR coupl* OR dyad* OR “family member” OR husband* OR “life partner” OR marital* OR married* OR mutuality OR partner* OR spous* OR wife OR wive)) AND (XB (stoma* OR enterostomy OR ostomy OR ileostomy OR colostomy) OR SU (stoma* OR enterostomy OR ostomy OR ileostomy OR colostomy)) AND (XB (“colorectal cancer” OR “CRC” OR “bowel cancer” OR “colon cancer” OR “rectal cancer”) OR SU (“colorectal cancer” OR “CRC” OR “bowel cancer” OR “colon cancer” OR “rectal cancer”)) AND (XB (Change* OR experience* OR “life-change event” OR “life changing event” OR “life-changing event” OR “life change*” OR transition* OR adjustment* OR adaptation) OR SU (Change* OR experience* OR “life-change event” OR “life changing event” OR “life-changing event” OR “life change*” OR transition* OR adjustment* OR adaptation))
PsycInfo (APA PsycNet)	(title: (caregiv*) OR title: (“care partner”) OR title: (conjugal) OR title: (coupl*) OR title: (dyad*) OR title: (“family member”) OR title: (husband*) OR title: (“life partner”) OR title: (marital*) OR title: (married*) OR title: (mutuality) OR title: (partner*) OR title: (spous*) OR title: (wife) OR title: (wive)) OR (abstract: (caregiv*) OR abstract: (“care partner”) OR abstract: (conjugal) OR abstract: (coupl*) OR abstract: (dyad*) OR abstract: (“family member”) OR abstract: (husband*) OR abstract: (“life partner”) OR abstract: (marital*) OR abstract: (married*) OR abstract: (mutuality) OR abstract: (partner*) OR abstract: (spous*) OR abstract: (wife) OR abstract: (wive)) OR (MeSH: (Caregivers) OR MeSH: (marriage) OR MeSH: (spouses))) AND ((title: (caregiv*) OR title: (“care partner”) OR title: (conjugal) OR title: (coupl*) OR title: (dyad*) OR title: (“family member”) OR title: (husband*) OR title: (“life partner”) OR title: (marital*) OR title: (married*) OR title: (mutuality) OR title: (partner*) OR title: (spous*) OR title: (wife) OR title: (wive)) OR (abstract: (caregiv*) OR abstract: (“care partner”) OR abstract: (conjugal) OR abstract: (coupl*) OR abstract: (dyad*) OR abstract: (“family member”) OR abstract: (husband*) OR abstract: (“life partner”) OR abstract: (marital*) OR abstract: (married*) OR abstract: (mutuality) OR abstract: (partner*) OR abstract: (spous*) OR abstract: (wife) OR abstract: (wive)) OR (MeSH: (Caregivers) OR MeSH: (marriage) OR MeSH: (spouses)) AND (title: (stoma* OR enterostomy OR ostomy OR ileostomy OR colostomy)) OR (abstract: (stoma* OR enterostomy OR ostomy OR ileostomy OR colostomy)) OR (MeSH: (Ostomy) OR MeSH: (“surgical stomas”)) AND (title: (“colorectal cancer”) OR title: (“CRC”) OR title: (“bowel cancer”) OR title: (“colon cancer”) OR title: (“rectal cancer”)) OR (abstract: (“colorectal cancer”) OR abstract: (“CRC”) OR abstract: (“bowel cancer”) OR abstract: (“colon cancer”) OR abstract: (“rectal cancer”)) OR (MeSH: (“intestinal neoplasms”) OR MeSH: (neoplasms)) AND (title: (Change*) OR title: (experience*) OR title: (“life-change event”) OR title: (“life changing event”) OR title: (“life-changing event”) OR title: (“life change*”) OR title: (transition*) OR title: (adjustment*) OR title: (adaptation)) OR (abstract: (Change*) OR abstract: (experience*) OR abstract: (“life-change event”) OR abstract: (“life changing event”) OR abstract: (“life-changing event”) OR abstract: (“life change*”) OR abstract: (transition*) OR abstract: (adjustment*) OR abstract: (adaptation)) OR (MeSH: (“life change events”) OR MeSH: (“adaptation, psychological”) OR MeSH: (“social adjustment”))
Scopus	(TITLE-ABS-KEY (caregiv* OR “care partner” OR conjugal OR coupl* OR dyad* OR “family member” OR husband* OR “life partner” OR marital* OR married* OR mutuality OR partner* OR spous* OR wife OR wive )) AND ( TITLE-ABS-KEY ( stoma*)) AND (TITLE-ABS-KEY ( “colorectal cancer” OR “CRC” OR “bowel cancer” OR “colon cancer” OR “rectal cancer”)) AND (TITLE-ABS-KEY (Change* OR experience* OR “life-change event” OR “life changing event” OR “life-changing event” OR “life change*” OR transition* OR adjustment* OR adaptation))
*Web of Science Core Collection* (*Web of Science*)	(TS=(caregiv* OR “care partner” OR conjugal OR coupl* OR dyad* OR “family member” OR husband* OR “life partner” OR marital* OR married* OR mutuality OR partner* OR spous* OR wife OR wive)) AND (TS=(stoma* OR enterostomy OR ostomy OR ileostomy OR colostomy)) AND (TS=(“colorectal cancer” OR “CRC” OR “bowel cancer” OR “colon cancer” OR “rectal cancer”)) AND (TS=(Change* OR experience* OR “life-change event” OR “life changing event” OR “life-changing event” OR “life change*” OR transition* OR adjustment* OR adaptation))
SciELO *Citation Index* (*Web of Science*)	(TS=(caregiv* OR “care partner” OR conjugal OR coupl* OR dyad* OR “family member” OR husband* OR “life partner” OR marital* OR married* OR mutuality OR partner* OR spous* OR wife OR wive)) AND (TS=(stoma* OR enterostomy OR ostomy OR ileostomy OR colostomy)) AND (TS=(“colorectal cancer” OR “CRC” OR “bowel cancer” OR “colon cancer” OR “rectal cancer”)) AND (TS=(Change* OR experience* OR “life-change event” OR “life changing event” OR “life-changing event” OR “life change*” OR transition* OR adjustment* OR adaptation))
Cochrane	(caregiv* OR “care partner” OR conjugal OR coupl* OR dyad* OR “family member” OR husband* OR “life partner” OR marital* OR married* OR mutuality OR partner* OR spous* OR wife OR wive):ti,ab,kw *MeSH descriptor: [Caregivers] explode all trees* *MeSH descriptor: [Marriage] explode all trees* *MeSH descriptor: [Spouses] explode all trees* *#1 OR #2 OR #3 OR #4* (stoma* OR enterostomy OR ostomy OR ileostomy OR colostomy):ti,ab,kw *MeSH descriptor: [Ostomy] explode all trees* *MeSH descriptor: [Surgical Stomas] explode all trees* *#6 OR #7 OR #8* *(“colorectal cancer” OR “CRC” OR “bowel cancer” OR “colon cancer” OR “rectal cancer”):ti,ab,kw* *MeSH descriptor: [Intestinal Neoplasms] explode all trees* *MeSH descriptor: [Neoplasms] explode all trees* *#10 OR #11 OR #12* (Change* OR experience* OR “life-change event” OR “life changing event” OR “life-changing event” OR life NEXT change* OR transition* OR adjustment* OR adaptation):ti,ab,kw *MeSH descriptor: [Life Change Events] explode all trees* *MeSH descriptor: [Adaptation, Psychological] explode all trees* *MeSH descriptor: [Social Adjustment] explode all trees* *#14 OR #15 OR #16 OR #17* *#5 AND #9 AND #13 AND #18*
*ProQuest Dissertations & Theses Citation Index* (*Web of Science*)	(TS=(caregiv* OR “care partner” OR conjugal OR coupl* OR dyad* OR “family member” OR husband* OR “life partner” OR marital* OR married* OR mutuality OR partner* OR spous* OR wife OR wive)) AND (TS=(stoma* OR enterostomy OR ostomy OR ileostomy OR colostomy)) AND (TS=(“colorectal cancer” OR “CRC” OR “bowel cancer” OR “colon cancer” OR “rectal cancer”)) AND (TS=(Change* OR experience* OR “life-change event” OR “life changing event” OR “life-changing event” OR “life change*” OR transition* OR adjustment* OR adaptation))
RCAAP (EBSCO)	(TI (caregiv* OR “care partner” OR conjugal OR coupl* OR dyad* OR “family member” OR husband* OR “life partner” OR marital* OR married* OR mutuality OR partner* OR spous* OR wife OR wive) OR TI (caregiv* OR “care partner” OR conjugal OR coupl* OR dyad* OR “family member” OR husband* OR “life partner” OR marital* OR married* OR mutuality OR partner* OR spous* OR wife OR wive) OR SU (caregiv* OR “care partner” OR conjugal OR coupl* OR dyad* OR “family member” OR husband* OR “life partner” OR marital* OR married* OR mutuality OR partner* OR spous* OR wife OR wive)) AND (TI (stoma* OR enterostomy OR ostomy OR ileostomy OR colostomy) OR AB (stoma* OR enterostomy OR ostomy OR ileostomy OR colostomy) OR SU (stoma* OR enterostomy OR ostomy OR ileostomy OR colostomy)) AND (TI (“colorectal cancer” OR “CRC” OR “bowel cancer” OR “colon cancer” OR “rectal cancer”) OR AB (“colorectal cancer” OR “CRC” OR “bowel cancer” OR “colon cancer” OR “rectal cancer”) OR SU (“colorectal cancer” OR “CRC” OR “bowel cancer” OR “colon cancer” OR “rectal cancer”)) AND (TI (Change* OR experience* OR “life-change event” OR “life changing event” OR “life-changing event” OR “life change*” OR transition* OR adjustment* OR adaptation) OR AB (Change* OR experience* OR “life-change event” OR “life changing event” OR “life-changing event” OR “life change*” OR transition* OR adjustment* OR adaptation) OR SU (Change* OR experience* OR “life-change event” OR “life changing event” OR “life-changing event” OR “life change*” OR transition* OR adjustment* OR adaptation)) *Filters: Content Provider: RCAAP*
*JBI Evidence Syntesis*	stoma* OR enterostomy OR ostomy OR ileostomy OR colostomy
*Google Scholar*	*allintitle: stoma cancer experience spouse* *allintitle: stoma cancer transition spouse* *allintitle: stoma cancer adaptation spouse* *allintitle: stoma cancer adjustment spouse* *allintitle: ostomy cancer experience spouse* *allintitle: ostomy cancer transition spouse* *allintitle: ostomy cancer adaptation spouse* *allintitle: ostomy cancer adjustment spouse* *allintitle: ostomia cancro experiência casal* *allintitle: ostomia cancro transição casal* *allintitle: ostomia cancro adaptação casal* *allintitle: ostomia cancro ajustamento casal*
*Open Access Thesis and Dissertations*	abstract (caregiv* OR partner OR conjugal OR coupl* OR dyad* OR family OR husband* OR marital* OR married* OR spous* OR wife OR wive) AND abstract (stoma* OR enterostomy OR ostomy OR ileostomy OR colostomy) AND abstract (cancer* OR neoplasm*) AND abstract (Change* OR experience* OR transition* OR adjustment* OR adaptation) subject (caregiv* OR partner OR conjugal OR coupl* OR dyad* OR family OR husband* OR marital* OR married* OR spous* OR wife OR wive) AND subject (stoma* OR enterostomy OR ostomy OR ileostomy OR colostomy) AND subject (cancer* OR neoplasm*) AND subject (Change* OR experience* OR transition* OR adjustment* OR adaptation) title (caregiv* OR partner OR conjugal OR coupl* OR dyad* OR family OR husband* OR marital* OR married* OR spous* OR wife OR wive) AND title (stoma* OR enterostomy OR ostomy OR ileostomy OR colostomy) AND title (cancer* OR neoplasm*) AND title (Change* OR experience* OR transition* OR adjustment* OR adaptation)

Las referencias identificadas se compilaron y cargaron en la plataforma Rayyan®, y se eliminaron los registros duplicados, lo que dio como resultado un total de 733 documentos. El análisis de los títulos y resúmenes de los documentos fue realizado por dos revisores independientes utilizando el método *blind-one*. Las discrepancias fueron resueltas por un tercer revisor^(33,36)^.

Los documentos que cumplieron los criterios de inclusión tras el análisis del título y del resumen se recuperaron en su totalidad para su análisis detallado, y el texto completo fue leído de forma independiente por los revisores. El proceso de selección de los documentos se representa en el diagrama de flujo PRISMA-ScR (Figura 1), que ilustra de forma sistemática las etapas del proceso^(37)^, culminando en la selección de nueve documentos.

**Figura 1:** Diagrama de flujo de Preferred Reporting Items for Systematic Reviews and Meta-Analyses extension for Scoping Reviews (Elementos de información preferidos para revisiones sistemáticas y metaanálisis, extensión para revisiones exploratorias)^(37)^.

### Análisis y Tratamiento de Datos

La extracción y presentación de los datos extraídos de los estudios incluidos siguieron una estructura específicamente diseñada para esta revisión de alcance, en función del objetivo y las cuestiones de la revisión^(33,34)^. La extracción de los datos fue realizada por dos revisores independientes, y las divergencias se resolvieron con la ayuda de un tercer revisor. Se extrajeron los datos relacionados con el objetivo y las cuestiones de la revisión (Cuadro 2), como el título, el autor o autores, el año, el país, el objetivo, la metodología y los métodos del estudio, la población objetivo y los principales resultados. Los datos recopilados se analizaron mediante recuento de frecuencias, para caracterizar los documentos incluidos, y mediante análisis de contenido cualitativo descriptivo, para resumir las características de la experiencia de la pareja. Los resultados se presentan en un cuadro y mediante una descripción narrativa.

**Cuadro 2:** Presentación de los datos extraídos – Lisboa, Portugal, 2025.

	Autor(es)/año/país	Metodología y métodos de estudio	Población	Objetivo	Principales resultados
**Estudio 1**	Du, Chaiviboontha y Sumdaengrit/2024/China^(38)^	Paradigma cualitativo Fenomenología Entrevista	16 personas con ostomía (edades comprendidas entre los 18 y los 59 años)	Explorar las experiencias de los supervivientes de cáncer colorrectal en la intimidad conyugal tras someterse a una ostomía.	El estudio identificó cinco dimensiones fundamentales en la experiencia conyugal de los supervivientes de cáncer colorrectal con ostomía: intimidad física, psicológica, social, espiritual y operativa. La presencia del estoma afecta negativamente a la relación sexual, generando preocupaciones sobre la atractividad y la autoestima, lo que se refleja en una disminución de la libido y en dificultades para expresar afecto. Aun así, las conexiones emocionales y espirituales se perciben como particularmente significativas, contribuyendo a redefinir el sentido de identidad y autoestima. La gestión diaria (intimidad operativa) surge como un elemento crítico, que requiere colaboración, apoyo del cuidador y habilidades para la resolución de conflictos, lo que contribuye a una adaptación exitosa.
**Estudio 2**	Afiyanti, Fitch, Helen, Andjarwati, Rudi, Prawesti y Juliastuti/2023/Indonesia^(39)^	Paradigma cualitativo Fenomenología Entrevista	12 personas con ostomía (edades comprendidas entre 31 y 58 años)	Explorar la perspectiva de las personas que viven con una ostomía sobre sus experiencias sexuales y las influencias físicas, psicológicas y culturales.	El estudio puso de manifiesto trastornos sexuales significativos en los supervivientes de cáncer colorrectal con ostomía, entre ellos disminución del deseo, malestar e insatisfacción, lo que llevó a algunos a interrumpir por completo la intimidad sexual. El apoyo del cónyuge se reveló como un factor positivo, ya que potenciaba una mejor comunicación y una mayor disponibilidad para la convivencia, lo que los participantes reconocieron como beneficioso. Muchas estrategias de adaptación se centraron en el bienestar conyugal, a veces en detrimento de las necesidades sexuales individuales. Sin embargo, se observó una falta de comunicación sobre cuestiones sexuales con los profesionales de la salud. Los resultados subrayan la necesidad de apoyo interdisciplinario y de asesoramiento pre y posquirúrgico para mejorar la salud sexual y la relación conyugal.
**Estudio 3**	Du, Wang, Du, Zou y Jin/2021/China^(40)^	Paradigma cuantitativo Estudio correlacional Un cuestionario y tres escalas	390 personas con ostomía (el 59,2 % eran hombres y el 60,2 % de ellos tenían más de 60 años)	Evaluar la correlación entre la relación íntima con la pareja, la autorrevelación y la adaptabilidad en pacientes con cáncer colorrectal y enterostomía.	El estudio destaca la aceptación como la dimensión más valorada por los participantes. Por otro lado, la autorrevelación también se correlacionó positivamente con los niveles de adaptación, aceptación y una actitud positiva ante la vida, mientras que se correlacionó negativamente con la preocupación continua. La mayoría de los participantes (42,6 %) refirieron problemas en las relaciones íntimas conyugales, y el 90,8% presentaba niveles de adaptación bajos o moderados. Los predictores significativos para las relaciones íntimas conyugales incluyeron la formación académica, la capacidad de autocuidado y el tiempo postoperatorio. El estudio destaca la necesidad de la educación sanitaria para mejorar la autorrevelación y la adaptabilidad entre los pacientes con cáncer colorrectal.
**Estudio 4**	McCarthy/2020/Canadá^(41)^	Paradigma cualitativo Estudio 1: *teoría fundamentada* (entrevista semiestructurada a parejas) Solo el estudio 1 cumple los criterios de inclusión	11 parejas (portadores de ostomía con edades comprendidas entre 42 y 80 años; cónyuges con edades comprendidas entre 37 y 76 años)	Proporcionar un relato experiencial de la adaptación sexual e íntima de las parejas a una colostomía permanente (tras el tratamiento y el cáncer colorrectal).	Los resultados evidencian un sufrimiento emocional y preocupaciones identitarias significativas en las personas con cáncer colorrectal, lo que refuerza la importancia de los cuidados de apoyo proactivos por parte de los profesionales de la salud para mejorar su calidad de vida. A pesar del impacto de los tratamientos oncológicos y la ostomía en el funcionamiento sexual y la imagen corporal, las parejas demuestran su capacidad para permanecer unidas como díada y resistir los cambios provocados. Aunque algunos informan de un fortalecimiento de la intimidad, persisten los retos físicos y psicosociales en la adaptación sexual. Los resultados se presentaron en dos categorías. En la categoría “Amar con un cuerpo imperfecto después de una colostomía”, se destaca la importancia de la reconstrucción de la imagen corporal como facilitadora de la aceptación del cuerpo y la integración de la ostomía en su identidad, la (in)comodidad de la pareja y la influencia de la aceptación mutua en la apertura a la intimidad sexual. En la categoría “Lidiar con la función sexual después del cáncer”, se destacan las implicaciones de los tratamientos en la función y la identidad sexual, el ritmo individual de cada miembro de la pareja, la necesidad de pensar, de manera global, en la comunicación conjunta y la exploración de diferentes formas de intimidad.
**Estudio 5**	Tripaldi/2019/Irlanda^(42)^	Revisión de la literatura	18 artículos: dos revisiones de la literatura y el resto eran estudios de investigación (estudios cualitativos, cuantitativos y mixtos)	Explorar la experiencia sexual de las mujeres tras la formación de un estoma en el curso de una cirugía por cáncer colorrectal.	La revisión identificó tres temas principales —alteración de la imagen corporal, relaciones íntimas y desvalorización de la sexualidad—, lo que pone de manifiesto la insuficiencia de la educación preoperatoria en el ámbito de la salud sexual. Las mujeres afirmaron sentirse desprevenidas ante el impacto del estoma, enfrentándose a dificultades en la función sexual y en la aceptación por parte de la pareja. La presencia del estoma acentúa los cambios en la imagen corporal, contribuyendo a una reducción de la autoestima y l e de la percepción de la atractividad sexual asociada a la pérdida de libido, sequedad vaginal y limitaciones en la participación en relaciones sexuales con penetración. Las mujeres tienden a anticipar la aceptación o el rechazo por parte de su pareja, y también se han descrito dificultades conyugales. El apoyo de la pareja resultó ser esencial para la adaptación de las mujeres, aunque no todas las parejas lograron ofrecer un apoyo emocional adecuado. Por lo tanto, resulta evidente la importancia de una intervención educativa y psicosocial integrada en el período posoperatorio como estrategia fundamental para promover el bienestar de estas mujeres.
**Estudio 6**	Alwi, Setiawanb & Asrizalb/2018/Indonesia^(43)^	Paradigma cualitativo Fenomenología Entrevista	12 personas con ostomía	Describir las experiencias de las personas con colostomía definitiva en relación con su calidad de vida.	El estudio identificó siete temas centrales en la calidad de vida de las personas con colostomía permanente, a saber: limitaciones en las actividades de la vida diaria; restricciones en las relaciones conyugales y sociales; sentimientos negativos hacia la colostomía; dificultades económicas; exigencias crecientes de la vida; cambios en las necesidades de descanso y complicaciones físicas; y esperanza de llevar una vida normal después de la colostomía. En particular, las relaciones conyugales pusieron de manifiesto preocupaciones relacionadas con el divorcio y el cese de las relaciones sexuales, lo que generó temor y abstinencia después de la cirugía. En la interacción social, surgieron como principales obstáculos la ansiedad, la privacidad, diversas dificultades prácticas y rasgos de personalidad introvertida.
**Estudio 7**	Purba, Ibrahim y Rahayu/2018/Indonesia^(44)^	Paradigma cualitativo Fenomenología Entrevista	Tres personas con ostomía (edad entre 40 y 50 años)	Obtener la experiencia significativa de los supervivientes de cáncer rectal.	El estudio evidencia que los supervivientes de cáncer rectal con estoma viven experiencias que afectan a la relación conyugal, sobre todo en las áreas de intimidad, sexualidad, apoyo mutuo, gestión financiera y pérdida. La autoestima y la imagen corporal se ven comprometidas, lo que genera vergüenza, aislamiento y dificultad para intimar con la pareja. En el plano sexual, todos informaron cambios en la actividad, marcados por el miedo al dolor, el rechazo o la incomodidad, lo que exige un mayor apoyo profesional sensible al contexto cultural. La pérdida de la integridad física y del futuro idealizado se afronta con tristeza, aceptación progresiva y necesidad de adaptación, a menudo respaldada por la fe. A pesar de las dificultades, destaca el deseo de ayudar a otras personas en situaciones similares, lo que contribuye al fortalecimiento emocional.
**Estudio 8**	McCarthy, Fergus y Miller/2016/Canadá^(45)^	Paradigma cualitativo (análisis temático teórico) Entrevista	Nueve parejas (edades comprendidas entre 42 y 80 años)	Validar el Sistema de Clasificación de Adaptación de la Pareja al Cáncer utilizando una muestra de parejas que se han adaptado al cáncer rectal y a la colostomía permanente, y mejorar la estructura basándose en los datos emergentes de la muestra.	El estudio constató que todas las parejas experimentaron fluctuaciones entre el “yo” y el “nosotros” durante el proceso de adaptación al cáncer de recto y a la colostomía, poniendo de manifiesto diferentes formas de afirmación y diferenciación del “nosotros”. Mientras que algunos participantes identificaron cambios claros en la relación y estuvieron de acuerdo con la conceptualización de que existe una variación continua entre la unión y la separación, otros manifestaron una reacción más ambigua, aunque reconocieron la necesidad de espacio físico y emocional a lo largo del curso de la enfermedad. Aunque el respeto por las necesidades individuales no es algo nuevo en la dinámica relacional, varias parejas destacaron ajustes específicos relacionados con las actividades de la colostomía y el tratamiento del cáncer, como la reorganización de las responsabilidades prácticas. Además, algunas parejas relataron momentos de mayor cercanía y empatía, reconociendo que estas fases de “unión” pueden alternarse con períodos de distanciamiento o aislamiento, sin que ello perjudique de forma definitiva el vínculo conyugal. Por el contrario, el reconocimiento mutuo de la independencia y la autonomía resultó fundamental para fortalecer el funcionamiento colectivo e individual. En resumen, las dinámicas de afirmación y erosión del “nosotros”, evidentes a lo largo de la adaptación a la enfermedad, resultan naturales y no constituyen necesariamente un indicador de fracaso relacional. Por el contrario, superar juntos estos retos refuerza la resiliencia de la pareja, intensificando el intercambio, la empatía y una mayor identificación con la relación.
**Estudio 9**	Persson, Severinsson y Hellström/2004/Suecia^(46)^	Paradigma cualitativo (análisis de contenido) Entrevista grupal	Nueve cónyuges de personas con ostomía (seis de ellos mujeres, con edades comprendidas entre los 49 y los 74 años)	Conocer las percepciones de los cónyuges sobre cómo viven con una pareja a la que se le ha diagnosticado cáncer de recto seguido de una cirugía que ha dado lugar a una ostomía.	Los principales resultados del estudio revelan que la experiencia de los cónyuges de personas sometidas a ostomía por cáncer colorrectal se caracteriza por una serie de retos. Los cónyuges, aunque intentan apoyar a sus parejas, informan de dificultades para involucrarse en los cuidados y en el proceso de información, sintiéndose a menudo excluidos y emocionalmente solos, ya que la atención se centra exclusivamente en el paciente. La incertidumbre sobre la evolución de la enfermedad y el temor a la recurrencia del cáncer generaban una ansiedad constante, agravada por la falta de información y la percepción de no estar incluidos en las decisiones clínicas. Después de la cirugía, fue necesario aprender a vivir de una nueva forma, reorganizando la rutina familiar, los hábitos sociales y las estrategias de cuidado en función de las exigencias del estoma, lo que implicó un ajuste familiar y una adaptación a la nueva condición. La presencia del estoma tuvo un impacto significativo en la imagen corporal y la intimidad de la pareja, afectando a la sexualidad y generando sentimientos ambivalentes, como repulsión y culpa. Por último, los cónyuges buscaban explicaciones al origen de la enfermedad, tratando de encontrarle sentido al sufrimiento mediante el análisis de factores hereditarios o del estilo de vida de la pareja.

## RESULTADOS

Se identificaron 733 referencias mediante una búsqueda electrónica en bases de datos y repositorios. De ellas, 105 se encontraron en la base de datos MEDLINE, 73 en CINAHL, cuatro en SciELO, nueve en *JBI Evidence Synthesis*, dos en *Psychology and Behavioral Sciences Collection*, 19 en PsycINFO, 105 en *Web of Science*, 356 en Scopus, 37 en Cochrane, ocho en *Open Access Theses and Dissertations*, 13 en *ProQuest Dissertation and Theses Database* y dos en RCAAP. La búsqueda realizada en *Google Scholar* no arrojó resultados relevantes.

De los 733 registros identificados, 710 procedían de las bases de datos y 23 eran resultado de otros métodos de búsqueda. En cuanto a los registros identificados en las bases de datos, 212 fueron excluidos por ser duplicados. De los 498 registros restantes, 463 se eliminaron tras leer el título y el resumen, y se seleccionaron 35 para su recuperación en texto completo. De estos, se excluyó un registro debido a la imposibilidad de acceder al texto completo, a pesar de los esfuerzos realizados en este sentido. La lectura íntegra de los 34 registros restantes dio lugar a la exclusión de 26, por no cumplir los criterios de inclusión, en particular porque la ostomía no se debía a una cirugía por CCR, porque la población incluía simultáneamente a personas con y sin ostomía, porque la población no correspondía a la pareja o porque abordaban un concepto distinto. En cuanto a los registros identificados por otros métodos, tras leer el título y el resumen, se evaluaron tres según los criterios de inclusión, y se excluyeron dos porque la ostomía no se debía a una cirugía por CCR y porque la población incluía personas con y sin ostomía. Así, se incluyó en la revisión un estudio procedente de otros métodos. En total, se incluyeron en esta revisión nueve estudios (Figura 1).

Los estudios incluidos se publicaron entre 2004 y 2024, con al menos una publicación al año entre 2019 y 2024^(38,39,40,41,42)^, a excepción de 2022, en el que no se registraron publicaciones. También se verificaron dos publicaciones en 2018^(43,44)^y una publicación en los años 2016^(45)^y 2004^(46)^. En cuanto al origen geográfico de los estudios, se observó la existencia de tres estudios realizados en Indonesia^(39,43,44)^, dos en Canadá^(41,45)^, dos en China^(38,40)^, uno en Irlanda^(42)^ y uno en Suecia^(46)^. En cuanto al tipo de estudios incluidos, siete se enmarcan en el paradigma cualitativo, cuatro de ellos recurriendo al enfoque fenomenológico^(38,39,43,44)^y uno a la *Grounded Theory* ^(41)^, mientras que uno utiliza el análisis temático de orientación teórica^(45)^y otro el análisis de contenido^(46)^. Uno de los estudios se inscribe en el paradigma cuantitativo, de naturaleza correlacional^(40)^, y otro corresponde a un estudio secundario, en forma de revisión de la literatura^(42)^. En cuanto a la población, cinco estudios incluyeron exclusivamente a personas con ostomía^(38,39,40,43,44)^, y dos incluyeron a parejas^(41,45)^, en las que uno de los miembros era portador de ostomía, y uno incluyó a cónyuges de personas con ostomía^(46)^. Aunque los criterios de elegibilidad definidos consideraban adultos a las personas de 19 años o más, algunos estudios consideran adultos a las personas de 18 años o más. Así, la edad de los participantes osciló entre los 18 y los 80 años.

En cuanto a los conceptos utilizados en la evidencia consultada para describir la experiencia de la pareja ante una OEI por CCR, se identificaron los conceptos de adaptación y ajuste.

Se realizó un análisis de contenido cualitativo de naturaleza descriptiva de los resultados de los estudios incluidos. Se llevó a cabo una lectura minuciosa e iterativa de los datos, a partir de la cual se identificaron, de forma inductiva, los temas que caracterizaban la experiencia de la pareja. Posteriormente, estos temas se agruparon, por similitud, en categorías y subcategorías. El proceso de análisis se llevó a cabo manualmente, sin recurrir a software específico de apoyo al análisis cualitativo, y se discutió entre los investigadores hasta alcanzar un consenso sobre la definición y la organización final de las categorías.

El análisis de los datos extraídos permitió caracterizar la experiencia de la pareja que se enfrenta a una OEI por CCR en tres categorías: naturaleza de la experiencia; factores influyentes; y resultados derivados de esa experiencia (Cuadro 3).

**Cuadro 3:** Caracterización de la experiencia de la pareja – Lisboa, Portugal, 2025.

Dimensiones		Datos
**Naturaleza** **de la experiencia**		Desafío constante al lidiar con preocupaciones físicas y psicosociales nuevas y antiguas^(41,46)^ Sentimiento de desconexión “en el mismo espacio, pero sin conexión” con su pareja^(45)^ Fluctuación entre el “yo” y el “nosotros”^(45)^ Inevitable aislamiento del cónyuge en determinados momentos^(45)^ Varios procesos de afirmación y diferenciación del “nosotros”^(45)^
**Factores**	**Facilitadores**	Apoyo interdisciplinario^(38–40,42)^ y asesoramiento pre y posquirúrgico^(39,40,42)^ Cuidados colaborativos^(38,39,45,46)^ y redistribución de responsabilidades^(45,46)^ Apoyo/respaldo del cónyuge^(39,41,42,46)^ Cuidados de apoyo por parte de los profesionales sanitarios^(41)^ Capacidad de autocuidado^(40,42)^ Comunicación en la pareja^(39,41)^ y en la sociedad (comunidad)^(41)^ Pensar en términos globales^(41)^ Definición de objetivos comunes, reconocimiento sincero y ánimo^(38,46)^ Información, acogida por parte de los profesionales sanitarios y su capacidad de respuesta^(44)^ Actitud positiva ante la vida^(40,46)^ Esperanza de llevar una vida normal tras la ostomía^(43,46)^ Confianza mutua^(38)^ Comprensión mutua, empatía y experiencia interpersonal^(45)^ Recurrir al sentido de “nosotros” para superar la adversidad^(45)^ Contacto físico no sexual^(38)^ Contacto con la naturaleza^(38)^ Condición socioeconómica estable^(41)^ Fe^(38)^
	**Inhibidores**	Sentimientos negativos hacia la ostomía, como miedo^(39,43,44,46)^, inseguridad^(43,44,46)^, ansiedad^(38,43,46)^, depresión^(38)^, vergüenza^(38,43)^, preocupación^(38,40–44)^, estrés psicológico^(38,42)^ Falta de información y educación sanitaria^(38,40–42,46)^ Disminución de la libido^(38,39,42,43,46)^ Disminución del atractivo^(38,42,44,46)^ Falta de comunicación y apoyo de los profesionales^(39,42)^ Falta de interés por parte de la pareja^(38)^ Dificultades económicas^(43, 44)^
**Resultados**	**Positivos**	Aceptación de la nueva situación^(38,40–42,45,46)^ Mejora de la comunicación en la pareja^(38,39,41–43)^ y con la sociedad/comunidad^(41)^ Apoyo e interacción conyugal y social^(38,41,42,45,46)^ Cuidados colaborativos entre la pareja^(38,39,45,46)^ Capacidad de resistencia a los cambios sexuales^(41)^ Desarrollo como pareja^(39,41,45)^ Sentimiento de unión^(41,46)^ Acercarse más el uno al otro^(39,45)^ Afrontar y reparar la autoimagen^(38,41,46)^ Afrontar el impacto de la ostomía y el tratamiento oncológico en la relación sexual^(45)^ Reformulación de la identidad tras la ostomía^(41)^ Preservación de la identidad del “yo” por parte del otro^(41)^ Celebración y ampliación del repertorio sexual^(41)^ Refuerzo de los vínculos emocionales y espirituales en detrimento de la intimidad sexual^(38)^ Curación espiritual^(38)^ Redescubrir el valor personal y el significado de la vida^(38)^
	**Negativos**	Limitaciones en las actividades cotidianas, en las relaciones conyugales^(38,41,43,44)^ y en la vida sexual^(38,39,42–44)^ Cambios en las necesidades físicas y de descanso^(42–44)^ Cambios en el estilo y los patrones sexuales^(39,42,44,46)^ Desgaste en la relación^(38,42,46)^ Aislamiento social^(38,43,44,46)^ Baja satisfacción^(38,39,44)^ y baja autoestima^(42,44,46)^ Cambio de identidad^(38)^ Sufrimiento emocional significativo^(41)^

En cuanto a la naturaleza de la experiencia, los estudios analizados describen una serie de retos y transformaciones vividos por las parejas. Se ha informado de que esta experiencia supone un reto constante, marcado por la convivencia simultánea con preocupaciones físicas y psicosociales tanto recientes como anteriores^(41,46)^. Se identificaron sentimientos de desconexión entre los cónyuges, expresados por la percepción de “estar en el mismo espacio, pero sin estar conectados”^(45)^, así como una fluctuación entre la individualidad (“yo”) y la identidad relacional (“nosotros”)^(45)^. También se han descrito procesos de diferenciación y afirmación del “nosotros” conyugal^(45)^, así como la inevitable aparición de períodos de aislamiento entre los miembros de la pareja^(45)^.

Entre los factores que facilitan el proceso de adaptación, destacan el apoyo interdisciplinario^(38,39,40,42)^ y el asesoramiento pre y posquirúrgico^(39,40,42)^. La prestación de cuidados colaborativos^(38,39,45,46)^ y la redistribución de responsabilidades dentro de la pareja^(45,46)^ también fueron relevantes. El apoyo del cónyuge^(39,41,42,46)^ y la atención de apoyo prestada por los profesionales de la salud^(41)^ surgieron como determinantes, a partir de la capacidad de autocuidado^(40,42)^, la comunicación conyugal^(39,41)^ y la interacción en la comunidad^(41)^. Otros elementos considerados facilitadores fueron el pensamiento global^(41)^, la definición de objetivos conjuntos y el estímulo mutuo^(38,46)^, la disponibilidad de información y la acogida por parte de los profesionales de la salud^(44)^, así como una actitud positiva ante la vida^(40,46)^, la esperanza de retomar una vida normal tras la ostomía^(43,46)^, la confianza mutua^(38)^, la empatía y la comprensión interpersonal^(45)^, el recurso al sentido de “nosotros” para superar la adversidad^(45)^, el contacto físico no sexual^(38)^, el contacto con la naturaleza^(38)^, la condición socioeconómica estable^(41)^y la fe^(38)^. Por otro lado, como factores inhibidores, se destacaron los sentimientos negativos asociados a la ostomía, como el miedo^(39,43,44,46)^, la inseguridad^(43,44,46)^, la ansiedad^(38,43,46)^, la depresión^(38)^, la vergüenza^(38,43)^, la preocupación^(38,40,41,42,43,44)^ y el estrés psicológico^(38,42)^. La falta de información y educación para la salud^(38,40,41,42,46)^, la disminución de la libido^(38,39,40)^ y la falta de comunicación con la pareja^(38,40)^ también se mencionaron como factores inhibidores. y estrés psicológico^(38,42)^. La falta de información y educación para la salud^(38,40,41,42,46)^, la disminución de la libido^(38,39,42,43,46)^, la percepción de disminución del atractivo^(38,42,44,46)^, la falta de comunicación y apoyo por parte de los profesionales de la salud^(39,42)^, la falta de interés por parte de la pareja^(38)^y las dificultades económicas^(43,44)^también se identificaron como barreras importantes para el proceso de adaptación.

El análisis de los estudios también permite identificar una serie de resultados tanto positivos como negativos. En cuanto a los resultados positivos, se informó de la aceptación de la nueva condición de vida^(38,40,41,42,45,46)^, la mejora de la comunicación entre los miembros de la pareja^(38,39,41,42,43)^ y con la comunidad^(41)^, el refuerzo del apoyo conyugal y social^(38,41,42,45,46)^ y la implementación de cuidados colaborativos dentro de la relación^(38,39,45,46)^. También se observó la capacidad de resistencia a los cambios en la vida sexual^(41)^, el desarrollo como pareja^(39,41,45)^, el fortalecimiento del sentimiento de unión^(41,46)^y la percepción de una mayor cercanía entre los miembros de la pareja^(39,45)^. Asimismo, se identificaron procesos de afrontamiento y reparación de la autoimagen^(38,41,46)^, de superación de los impactos de la ostomía y del tratamiento oncológico en la sexualidad^(45)^, de reformulación de la identidad tras la ostomía^(41)^ y de preservación de la identidad del “yo” por parte del otro^(41)^. Además, se mencionaron experiencias de celebración y expansión del repertorio sexual^(41)^, refuerzo de los vínculos emocionales y espirituales en detrimento de la intimidad sexual^(38)^, curación espiritual^(38)^y redescubrimiento del valor personal y del significado de la vida^(38)^. Por último, en cuanto a los resultados negativos, se mencionaron limitaciones en las actividades de la vida diaria, en las relaciones conyugales^(38,41,43,44)^ y en la vida sexual^(38,39,42,43,44)^, cambios en las necesidades físicas y de descanso^(42,43,44)^, así como modificaciones en el estilo y el patrón sexual^(39,42,44,46)^. También se identificaron indicios de desgaste relacional^(38,42,46)^, aislamiento social^(38,43,44,46)^, baja satisfacción conyugal^(38,39,44)^, baja autoestima^(42,44,46)^, alteraciones de la identidad^(38)^ y sufrimiento emocional significativo^(41)^. Además, las lagunas identificadas en la evidencia se refieren a la escasez de estudios primarios que: i) se dediquen a la experiencia de la pareja ante una OEI por CCR; ii) incluyan como participantes a la díada conyugal; iii) describan la dinámica de la experiencia de estas parejas, en particular los procesos de cambio vividos; y iv) aborden la experiencia de la pareja como un proceso de transición múltiple —de salud/enfermedad y situacional— vivido por la pareja.

## DISCUSIÓN

Los resultados reflejan la existencia de un número reducido de estudios sobre la experiencia de la pareja que se enfrenta a una OEI por CCR, destacando los estudios que se centran en la sexualidad de la pareja (cinco de los nueve estudios)^(38,39,40,41,42)^, privilegiando, en la mayoría de los casos, la perspectiva individual. Esta escasez de estudios denota que este fenómeno aún está poco estudiado, lo que apunta a la necesidad de desarrollar estudios primarios. En la evidencia consultada, solo dos estudios contaron con la participación directa de la pareja^(41,45)^, mientras que los demás obtuvieron los datos sobre la experiencia de la pareja a través de la persona ostomizada^(38,39,40,43,44)^o del cónyuge^(46)^. Esta opción metodológica puede comprometer la comprensión de la experiencia conyugal de la OEI por CCR, ya que los fenómenos relacionales son, por naturaleza, interdependientes. Como señalan Revenson *et al.* ^(47)^, los fenómenos con un alto impacto funcional y simbólico, como una ostomía derivada de CCR, deben entenderse como experiencias compartidas, lo que exige enfoques metodológicos capaces de captar la reciprocidad de los significados, las emociones y las estrategias de afrontamiento en el seno de la díada conyugal. En este sentido, varios autores^(48,49,50)^subrayan que el uso de datos diádicos permite acceder de forma más fiable a la complejidad de la relación conyugal. Cuando solo uno de los miembros de la pareja se incluye en el estudio, los datos obtenidos pueden reflejar percepciones parciales, sesgadas por factores emocionales, limitaciones narrativas o dificultades para expresar experiencias relacionales de forma exhaustiva.

El análisis de los objetivos de los estudios incluidos en esta revisión de alcance permitió verificar que, en general, estos se centran en la exploración de la experiencia de la pareja ante el impacto de la ostomía asociada a la CCR, con especial énfasis en la sexualidad de la pareja, la calidad de vida y los retos relacionados con la adaptación y el ajuste a la nueva condición. Los estudios incluidos reconocen diversos cambios experimentados por las parejas que viven con una OEI derivada de la CCR, concretamente en las dimensiones física, emocional, relacional, social y espiritual.

Los estudios que integran esta revisión de alcance utilizan los conceptos de adaptación y ajuste cuando se refieren a la experiencia. Aunque se utilizan con frecuencia, estos conceptos rara vez van acompañados de definiciones, lo que dificulta su comprensión, distinción analítica y comparación entre estudios. No obstante, sobre la base del análisis realizado, se deduce que la adaptación es multidimensional^(38,41,42,45)^e implica la respuesta y la integración a las nuevas circunstancias. Es indispensable para mantener el bienestar emocional, físico y psicológico de la pareja,así como la calidad de la relación conyugal^(38,41,42,45)^. Por su parte, el ajuste corresponde a una trayectoria continua de respuesta práctica a los cambios físicos, psicológicos y sociales impuestos por la condición^(39,43,45)^. En general, el ajuste se describe como las estrategias adoptadas para lidiar con las alteraciones sexuales después de la cirugía^(39)^, los cambios realizados en las rutinas diarias, el cuidado personal y las interacciones sociales^(43)^, y desde una perspectiva relacional, implica un delicado equilibrio entre la preservación de la identidad individual de cada elemento y el mantenimiento del vínculo afectivo y funcional de la pareja^(45)^. La evidencia disponible permite percibir que estos conceptos están interrelacionados. En el estudio de Tripaldi^(42)^, los conceptos de adaptación y ajuste están presentes de forma implícita, pero no se presentan de forma independiente ni se diferencian conceptualmente, sino que aparecen integrados en la discusión de estrategias de afrontamiento, cambios en la identidad y dinámicas relacionales. Sin embargo, un análisis más detallado revela que la adaptación se concibe como una respuesta —que engloba la integración y la aceptación^(40,43)^—, mientras que el ajuste refleja un proceso activo y dinámico de cambio y negociación^(45)^.

Cabe destacar también que ninguno de los estudios recurrió explícitamente al concepto de transición para designar la experiencia vivida por la pareja que se enfrenta a una OEI en el contexto del CCR. Dado que la transición se define como un proceso y resultado del paso de una condición o estado a otro, en particular los relacionados con la salud y el desempeño de roles^(24,25)^, sería de esperar que dicho concepto se utilizara en la investigación de la experiencia de estas parejas. Esta ausencia pone de manifiesto una fragilidad relevante en el enfoque teórico de la problemática en estudio y sugiere la pertinencia de profundizar en la comprensión de esta experiencia a la luz de la perspectiva de la Teoría de las Transiciones, contribuyendo así a un análisis más amplio y sensible a las dinámicas procesales involucradas.

En relación con la naturaleza de la experiencia, los estudios destacan los retos y los cambios que experimentan las parejas. Varios autores^(41,46)^ señalan que esta experiencia constituye un reto constante, ya que las parejas se enfrentan simultáneamente a preocupaciones emergentes relacionadas con la enfermedad y la ostomía, y a cuestiones psicosociales preexistentes, que pueden reactivarse o agravarse en el contexto de la adversidad. Sin embargo, revelan que la experiencia se vive de manera divergente entre las parejas estudiadas, oscilando entre sentimientos de desconexión conyugal^(45)^y refuerzo del vínculo^(39,45)^entre el “yo” y el “nosotros”, lo que revela la complejidad de la adaptación a una nueva identidad, condicionada por la ostomía y las repercusiones de la enfermedad.

La evidencia científica demuestra que esta alternancia entre desconexión y fortalecimiento conyugal^(21)^, en el contexto de una ostomía, no ocurre de forma aleatoria, sino que es el resultado de la compleja interacción entre múltiples factores relacionales. En este sentido, la comunicación eficaz asume un papel decisivo: las parejas que logran compartir emociones, negociar roles e intercambiar información con franqueza tienden a reforzar el sentimiento de pertenencia y unidad (“nosotros”)^(51)^. Estos autores identifican que la eficacia diádica, es decir, la confianza conjunta en la capacidad de afrontar el cáncer, se ve facilitada por una comunicación fluida, un interés mutuo en la información y estrategias de afrontamiento comunes, mientras que las barreras en esta comunicación dan lugar a una menor cohesión y un mayor sufrimiento emocional.

La alineación de las expectativas y percepciones entre los cónyuges también resulta fundamental. Qinet al.^(52)^ destacan que los enfoques congruentes en la percepción del estrés y en las respuestas adaptativas, en particular entre el miedo a la recidiva y el enfoque conjunto, contribuyen a niveles superiores de resiliencia y ajuste conyugal. Cuando las percepciones divergen, por ejemplo, cuando la persona ostomizada minimiza el impacto mientras que el cónyuge se siente abrumado, tiende a surgir una creciente sensación de desconexión y desajuste en la pareja.

Además, el apoyo social y el acceso a recursos profesionales, como los programas de afrontamiento para parejas con CCR, han demostrado tener un impacto positivo en la experiencia compartida. Las intervenciones estructuradas que promueven la comunicación del estrés y el manejo comunitario, así como las estrategias educativas destinadas a involucrar a ambos miembros de la pareja, se han asociado con mejoras en la satisfacción relacional y la salud mental individual^(51,53,54)^. En esta línea, y de acuerdo con los datos obtenidos en esta revisión, se deduce que factores como la buena comunicación, el reparto de responsabilidades, la sintonía emocional y el apoyo profesional adecuado favorecen el surgimiento de un renovado sentido del “nosotros”. Este sentido compartido permite a las parejas afrontar la ostomía y las repercusiones del CCR con mayor cohesión y ajuste mutuo. Por el contrario, los factores inhibidores apuntan a barreras para la adaptación, en particular sentimientos de vergüenza, miedo, ansiedad y depresión, falta de información, apoyo profesional y dificultades económicas.

Los resultados de la revisión, al identificar como factor facilitador de la experiencia el apoyo de los profesionales de la salud y, como factores inhibidores, la escasez de información y educación para la salud, así como la ausencia de una comunicación profesional de apoyo, parecen evidenciar la necesidad de que los profesionales —en particular los enfermeros— profundicen sus conocimientos sobre este fenómeno, a fin de fundamentar intervenciones que apoyen eficazmente a las parejas ante esta experiencia.

La lectura crítica de los resultados permite constatar que la experiencia de la pareja ante una OEI por CCR es a la vez transformadora y vulnerable, integrando dimensiones positivas, como la aceptación, el apoyo mutuo, la colaboración en los cuidados y el refuerzo de la identidad conyugal, y dimensiones negativas, como los cambios en la intimidad sexual, el aislamiento emocional y el impacto en la autoestima y la identidad individual. Esta coexistencia de polaridades sugiere que la experiencia de la ostomía por CCR, cuando se vive en el contexto conyugal, no es lineal ni unívoca, y confirma que afrontar una OEI por CCR trasciende los límites de la adaptación individual, constituyendo una experiencia relacional que implica la renegociación de roles y significados en el seno de la díada conyugal. Esta perspectiva encuentra apoyo en la literatura reciente. Lin *et al.* ^(55)^, por ejemplo, subrayan que los cambios funcionales y las perturbaciones psicológicas interfieren significativamente en la intimidad conyugal y en la percepción del valor personal, lo que refuerza la importancia de las intervenciones orientadas a la relación diádica. En este sentido, los datos de esta revisión evidencian que los resultados positivos y negativos de la experiencia conyugal coexisten e interactúan, lo que sustenta la necesidad de estrategias de intervención específicas centradas en la pareja, como el asesoramiento sexual y conyugal, los programas educativos para parejas y el apoyo emocional continuo, que favorecen los procesos de ajuste mutuo^(53,54)^.

### Limitaciones

Se identifican como limitaciones de esta revisión de alcance el haber integrado un número reducido de estudios que abordan la experiencia de la pareja que se enfrenta a una OEI por CCR como unidad. Aunque la evidencia privilegia la experiencia de la pareja, el hecho de que la mayoría de los estudios seleccionados solo cuenten con un miembro ostomizado como participante puede haber generado cierto sesgo de percepción en los resultados de dichos estudios y, por lo tanto, también en esta revisión. A esto se suma una limitación de orden metodológico, relacionada con la imposibilidad de acceder al texto completo de uno de los artículos identificados durante el proceso de selección. A pesar de los esfuerzos, en particular la consulta de repositorios institucionales, bibliotecas académicas y el contacto directo con los autores, no fue posible garantizar su acceso, lo que restringió el conjunto de evidencias disponibles para el análisis.

### Contribuciones para el Área de Enfermería

Aunque la naturaleza de esta revisión no permite extraer implicaciones para la práctica, los resultados apuntan a elementos importantes que pueden orientar a los profesionales de la salud, concretamente a los enfermeros. Los resultados de esta revisión de alcance alertan sobre las exigencias y transformaciones a las que pueden enfrentarse las parejas en esta situación, poniendo de manifiesto la importancia de garantizar un apoyo continuo y una educación sanitaria mediante intervenciones que faciliten los procesos de cambio a los que se enfrenta la pareja, promoviendo, entre otros resultados, la resiliencia y el bienestar conyugal.

## CONCLUSIÓN

La presente revisión, sin límite temporal, permite concluir que hay escasa evidencia científica sobre el fenómeno. La evidencia disponible proviene de seis países. Aunque se refiere a la experiencia de la pareja ante una OEI por CCR, los participantes fueron en su mayoría personas con ostomía. Al nombrar esa experiencia, se destacó el recurso a los conceptos de adaptación y ajuste. La experiencia de estas parejas se caracterizó por la necesidad de ajustes continuos y mutuos ante los cambios impuestos por la ostomía y por una dualidad entre retos y oportunidades de crecimiento. Dicha experiencia se caracterizó en tres categorías: i) la naturaleza de la experiencia; ii) factores facilitadores e inhibidores; y iii) resultados positivos y negativos.

Centrándonos en la construcción de conocimientos relevantes para la enfermería, consideramos importante el desarrollo de una investigación primaria que permita mejorar la comprensión de la experiencia de las parejas que se enfrentan a la OEI por CCR. En este contexto, destaca la relevancia del estudio de la dinámica subyacente a su adaptación, ajuste o transición ante tales condiciones, es decir, el estudio del proceso o procesos de cambio a los que se enfrenta la díada. Los resultados de estos estudios pueden constituir una base de conocimientos para el desarrollo posterior de intervenciones que faciliten dichos procesos y la obtención de resultados en materia de salud para estas parejas.

## Data Availability

El conjunto de datos que respaldan las conclusiones de este estudio está disponible previa solicitud al autor correspondiente.
